# Human Gut-Commensalic *Lactobacillus ruminis* ATCC 25644 Displays Sortase-Assembled Surface Piliation: Phenotypic Characterization of Its Fimbrial Operon through *In Silico* Predictive Analysis and Recombinant Expression in *Lactococcus lactis*

**DOI:** 10.1371/journal.pone.0145718

**Published:** 2015-12-28

**Authors:** Xia Yu, Annukka Jaatinen, Johanna Rintahaka, Ulla Hynönen, Outi Lyytinen, Ravi Kant, Silja Åvall-Jääskeläinen, Ingemar von Ossowski, Airi Palva

**Affiliations:** Department of Veterinary Biosciences, Faculty of Veterinary Medicine, University of Helsinki, Helsinki, Finland; University of Illinois at Chicago College of Medicine, UNITED STATES

## Abstract

Sortase-dependent surface pili (or fimbriae) in Gram-positive bacteria are well documented as a key virulence factor for certain harmful opportunistic pathogens. However, it is only recently known that these multi-subunit protein appendages are also belonging to the “friendly” commensals and now, with this new perspective, they have come to be categorized as a niche-adaptation factor as well. In this regard, it was shown earlier that sortase-assembled piliation is a native fixture of two human intestinal commensalics (i.e., *Lactobacillus rhamnosus* and *Bifidobacterium bifidum*), and correspondingly where the pili involved have a significant role in cellular adhesion and immunomodulation processes. We now reveal that intestinal indigenous (or autochthonous) *Lactobacillus ruminis* is another surface-piliated commensal lactobacillar species. Heeding to *in silico* expectations, the predicted loci for the LrpCBA-called pili are organized tandemly in the *L*. *ruminis* genome as a canonical fimbrial operon, which then encodes for three pilin-proteins and a single C-type sortase enzyme. Through electron microscopic means, we showed that these pilus formations are a surface assemblage of tip, basal, and backbone pilin subunits (respectively named LrpC, LrpB, and LrpA) in *L*. *ruminis*, and also when expressed recombinantly in *Lactococcus lactis*. As well, by using the recombinant-piliated lactococci, we could define certain ecologically relevant phenotypic traits, such as the ability to adhere to extracellular matrix proteins and gut epithelial cells, but also to effectuate an induced dampening on Toll-like receptor 2 signaling and interleukin-8 responsiveness in immune-related cells. Within the context of the intestinal microcosm, by wielding such niche-advantageous cell-surface properties the LrpCBA pilus would undoubtedly have a requisite functional role in the colonization dynamics of *L*. *ruminis* indigeneity. Our study provides only the second description of a native-piliated *Lactobacillus* species, but at the same time also involves the structural and functional characterization of a third type of lactobacillar pilus.

## Introduction

Jutting outward from the cell periphery of certain Gram-positive bacteria are long proteinaceous structures dubbed sortase-dependent pili (or fimbriae). This sort of surface piliation retains a two- or three-subunit architecture, in which the covalent assembly of protein subunits (pilins) into a macromolecular form relies on the enzymatic action of so-called C-type sortases. Characteristically, the related pilus loci are clustered in the genome as an individual fimbrial operon, which includes genes for the pilin-proteins along with at least one sortase. During the ten-year interim between the initial confirmation of sortase-dependent piliation in the Gram-positives and now presently, most research was focused on pili from various pathogenic genera and species. Based on the numerous biochemical characterizations undertaken, the Gram-positive pathogen-derived pilus is viewed functionally relevant during cellular adhesion and host-cell colonization processes. For that reason, it is regarded as an identifiably significant virulence factor for a variety of disease-causing bacteria (for more detailed reviews, see [1,2]). However, over recent years, this perspective of Gram-positive surface piliation underwent a paradigm shift to include the more benign categorization of niche-adaptation factor after reports of pili in one particularly non-pathogenic species of lactic acid bacteria (LAB). Here, the pilus-like protrusions originally seen by Lebeer *et al* [3] on the cell surface of the human gut-commensalic (and probiotic) *Lactobacillus rhamnosus* GG strain were later established by us and others as representing prototypical sortase-assembled pili [4].

Predictably, *L*. *rhamnosus* GG pilus loci are arranged tandemly in the genome as an operon (so-called *spaCBA*) and include those for three pilin-proteins (*spaC*, *spaB*, and *spaA*) and an accompanying sortase enzyme (*srtC1*) [4]. When catalytically assembled, the SpaCBA-called pilus structure has a polymerized backbone of repeating SpaA subunits, on which to be found are ancillary pilins at the tip for adhesion (SpaC) and base for cell wall anchoring (SpaB) [4,5]. So far, the measured functionalities of the SpaCBA pilus include binding to mucus [6,7] and collagen [8], adhering to intestinal cells [9], mediating biofilm growth [9], provoking cellular responses in gut epithelial cells [10], and modulating host immune-cell responsiveness [9,11–13]. Besides the *spaCBA* operon, genes for another type of pilus (called SpaFED) can also be found in the *L*. *rhamnosus* GG genome [4], and similarly, they are clustered into a single operon (so-called *spaFED*) and encode three pilin subunits (*spaF*, *spaE*, and *spaD*) and one sortase (*srtC2*). Yet, unlike the *spaCBA* operon in *L*. *rhamnosus* GG, expression of loci from the fimbrial *spaFED* operon would appear to be constitutively silent in this strain [5]. Interestingly though, when the conjectured SpaFED pilus is produced recombinantly in *Lactococcus lactis*, its structure also takes on a prototypical subunit arrangement, with the tip-located SpaF pilin serving as the focal determinant for pilus-mediated cellular interactions with mucus, collagen, and fibronectin, and as well as the Caco-2 and HT-29 intestinal cell lines [14]. Although *L*. *rhamnosus* GG adaptation to the intestinal milieu reflects a transitory occupancy, this allochthonous colonizing behavior is considered comparatively less stringent than found with other non-piliated and less adherent strains [4,6]. Presumably, the SpaCBA pilus with its combined adhesive functionalities offers a distinct advantage and is undoubtedly one of the main reasons for *L*. *rhamnosus* GG cells having a relatively more protracted duration in the gut. As *L*. *rhamnosus* GG is often used probiotically, this could seemingly help prolong the advocated health benefits associated with this particular strain [15]. Still, while a piliated bacterium would likely have an improved niche-specific fitness and, in this case, making it more adhesively adaptable to certain environmental locales, the fimbrial *spaCBA* operon is itself a genomic rarity in the *L*. *rhamnosus* species [16].

Following the new evidence that revealed *L*. *rhamnosus* GG as a piliated strain [3,4], and then based on our unpublished observations (ca. 2009), we uncovered the genetic basis for sortase-dependent piliation in *Lactobacillus ruminis*, a species lying taxonomically outside the group of bacteria that includes the *L*. *rhamnosus* species. By using the NCBI database-deposited genome sequence of a human-derived intestinal isolate (ATCC 25644) we were able to identify the loci for three predicted pilin-proteins clustered in tandem with a single sortase. In all probability, these genes should encode for the assembly of a third lactobacillar pilus type, as the predicted primary structures of the corresponding subunits differ from the SpaCBA and SpaFED pilins. Shortly afterward, there came additional evidence from a published comparative genomics study of *L*. *ruminis* that then confirmed the clustered presence of pilus genes in a resequenced ATCC 25644 genome, but as well, in a newly sequenced genome from a strain of bovine gut origin (ATCC 27782) [17]. However, perhaps even more relatable, microarray-based observations of this fimbrial operon showed that the corresponding loci are actively transcribed in *L*. *ruminis* cells, with more up-regulated expression in the human strain than in the bovine isolate [17]. Ecologically, *L*. *ruminis* [18,19] is among the more dominating *Lactobacillus* species in the mammalian intestine [20], particularly in pigs [21]. Moreover, *L*. *ruminis* is one of the only few lactobacilli being recognized as an autochthonous (indigenous) part of the gut microbiota [22,23]. Whether such autochthony or indigeneity yields a true mutualistic relationship with the host intestine is still not fully known. Despite possessing a varied but interesting mix of phenotypic traits (i.e., broad host adaptability [17], obligate anaerobiosis [19], flagellar motility [24], and immunogenic responsiveness [25]), gut-dwelling *L*. *ruminis* has been scientifically sidelined for many years, which accounts for it being much less characterized and somewhat poorly understood in terms of molecular mechanisms and actions.

Given our research interests in lactobacillar surface piliation, we decided to investigate further the pilus operon of the human *L*. *ruminis* ATCC 25644 strain and, as a paradigm for study, then define its phenotypic features. Thus, for our present work, we *in silico* comparatively examined the primary structure of the predicted fimbrial proteins, and as well demonstrated their collective abilities to produce cell surface-assembled pili natively in *L*. *ruminis* and recombinantly in *L*. *lactis*. Moreover, by using recombinant piliated lactococci, we were able to pinpoint certain substrate binding specificities, and also resolve some possible molecular immunogenic effects imparted by this pilus type. Based on these obtained results, we regard that *L*. *ruminis* piliation is for the most part phenotypically analogous to the other types of sortase-dependent pili found in pathogenic and commensalic bacteria. However, more aptly, this pilus form exemplifies an advantageous surface-binding functionality that might favorably promote the autochthonous colonization dynamics associated with *L*. *ruminis* residency within the mammalian gut microcosm.

## Results and Discussion

### Genetic organization of the *L*. *ruminis* (ATCC 25644) fimbrial operon

**Fig 1:**
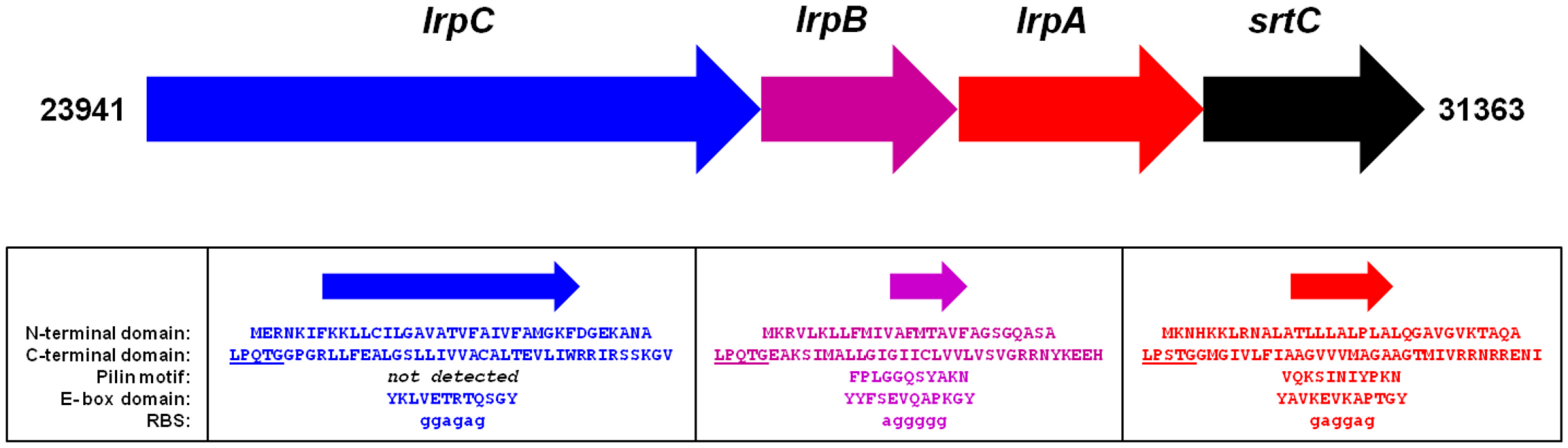
Schematic representation of the coding regions of the fimbrial *LrpCBA* operon from *L*. *ruminis* ATCC 25644. The clustered genomic arrangement of the *lrpC*, *lrpB*, *lrpA*, and *srtC* genes within the *LrpCBA* pilus operon is shown. Numbers indicate the beginning and ending nucleotide (nt) positions of the coding region for the operon. Arrows for the genes point in the direction of transcription and translation predicted from the DNA. Shown for each gene is nt sequence that resembles the ribosomal binding site (RBS). Deduced amino acid sequences for the N-terminal secretion signal and C-terminal sortase recognition domains in the predicted LrpC, LrpB, and LrpA pilin-proteins are provided. Residues for the LPXTG-like motifs in each pilin-protein are underlined. For each pilin-protein, predicted residues sharing similarity with the so-called pilin motif (WxxxVxVYPKN) and E-box domain (YxLxETxAPxGY) are indicated.

We had sourced the genome sequence information of the *L*. *ruminis* ATCC 25644 strain from the NCBI database (BioProjects: PRJNA55509 and PRJNA31499) and used it to identify the presence of one sortase-dependent pilus gene cluster in this *Lactobacillus* species. As depicted in Fig 1, for this particular human gut isolate the corresponding fimbrial operon extends from nucleotide (nt) position 23941 through to position 31363, with a genetic organization in the genome that entails open reading frames (ORFs) for the predicted tip, basal, and backbone pilin-proteins and one C-type sortase enzyme. Importantly, also existing for each of these ORFs is the upstream sequence potential for a typical ribosomal binding site (RBS) motif (5'-AGGAGG-3'), but as well, there is the absence of any premature stop codons, which then suggests this fimbrial operon can likely represent a functioning translational unit. From here on, we denote the *L*. *r* *uminis* pilus operon as *lrpCBA*, and of therein designate the pilin loci as *lrpC* (tip), *lrpB* (basal), and *lrpA* (backbone) and the pilin-specific sortase gene as *srtC*.

Our primary structure examination of the *lrpA*, *lrpB*, and *lrpC* loci from *L*. *ruminis* ATCC 25644 indicates each contains the sequence hallmarks of being prototypical Gram-positive pilin-proteins, such as having present what resembles the conserved amino acid pattern of the so-called pilin motif (WxxxVxVYPKN) and E-box domain (YxLxETxAPxGY) (Fig 1). Other pilin-characteristic features include the canonical domains involved in N-terminal secretion signaling (predicted by using the SignalP 4.1 server [26] at http://www.cbs.dtu.dk/services/SignalP/) and C-terminal sortase recognition, the latter sequence having an identifiable LPXTG-like motif at the beginning, with several aliphatic amino acids then following and punctuated by a few positive-charged residues at the end (Fig 1). Moreover, primary structural alignments of the predicted *lrpCBA*-encoded proteins in the human ATCC 25644 strain with those counterparts from bovine (ATCC 27782) [17], equine (DPC 6832; from the NCIB database), and porcine (our unpublished strain called GRL1172) isolates, but as well from another human strain (SPM0211) [27], had revealed a number of residue differences, with some of these being interspersed at certain positions along the sequence (S1 Fig). However, as might be anticipated from strains of the same species, amino acid identity among the various *lrpCBA*-encoded proteins is in fact relatively high, ranging from 89.7 to 99.8% for LrpC, 94.7 to 98.2% for LrpB, 90.3 to 100% for LrpA, and 96.9 to 100% for the C-type sortase (S1 Fig). Moreover, and as expected, the greatest degree of sequence similarity is seen with the alignment comparison data of the LrpCBA proteins from the two human strains (ATCC 25644 and SPM0211). More pointedly, it can be inferred from alignments between the primary structures of the predicted *lrpCBA*-encoded proteins and the corresponding proteins from the *L*. *rhamnosus* GG fimbrial *spaCBA* and *spaFED* operons (data not shown) that the LrpA, LrpB, and LrpC pilins would likely assemble into another type of *Lactobacillus* pilus (hereafter called LrpCBA). Here, we envisage that the multi-subunit architecture of an *lrpCBA*-encoded pilus consists of a polymerized LrpA-pilin backbone along with the LrpB and LrpC pilins at the base and tip, respectively.

Intriguingly, our protein sequence database searches for hidden Markov model (HMM) matches (done with Pfam [28] at http://pfam.xfam.org) using the LrpCBA pilins from the ATCC 25644 strain had revealed that among the three pilin subunits, it is only LrpC that shares homology with several protein domains explicitly identified as collagen binding (see further in S2 Fig). This is in contrast to the backbone LrpA and basal LrpB pilins, each of which is instead identified as exhibiting homology to one CnaB-type domain, whose presence is itself a structural feature universally shared by Gram-positive pilin-proteins (S2 Fig). From this, we anticipate that the predicted tip LrpC pilin will have a collagen-binding functionality, and thus might represent an adhesive determinant of the LrpCBA pilus. Our Pfam searches for other substrate-binding domains also revealed that none of the three LrpCBA pilins shares any similarities to those known for adherence to mucus. In fact, in our sequence analysis of the entire genome from *L*. *ruminis* ATCC 25644 we found no evidence of loci encoding proteins with resemblance to any familiar mucus-binding domains.

Interestingly as well, when using the LrpCBA pilin-proteins (see above) as sequence templates to perform a BlastP search of the NCIB database, we observed it is only the counterpart pilins from other *L*. *ruminis* strains that actually show a high level of amino acid identity (i.e., ≥ 91% for LrpC, ≥ 93% for LrpB, and ≥ 98% for LrpA). Unexpectedly, the next highest identities are not seen as any of the lactobacillar SpaCBA and SpaFED pilins. From this, we can interpret the *L*. *ruminis* fimbrial *lrpCBA* operon to be genome-specific to only one particular species, and thus quite unlike the *spaCBA* and *spaFED* pilus operons, which can be identified amongst a collection of three different yet related *Lactobacillus* species (*L*. *rhamnosus*, *L*. *casei*, and *L*. *paracasei*) [4,29–31]. Speculatively, when put in the natural context of the intestinal milieu, those *L*. *ruminis* strains with an active fimbrial *lrpCBA* operon will be better advantaged to outcompete and outlast non-appendage-bearing lactobacilli or any so-related commensalic bacteria. Taken more functionally, it is quite conceivable that the presence of this form of surface piliation and its accompanying physical outcome would embody a key facet in the autochthonous colonizing character of gut-dwelling *L*. *ruminis*.

### *In silico* insights into the upstream regulatory region of the *L*. *ruminis lrpCBA* pilus operon

**Fig 2:**
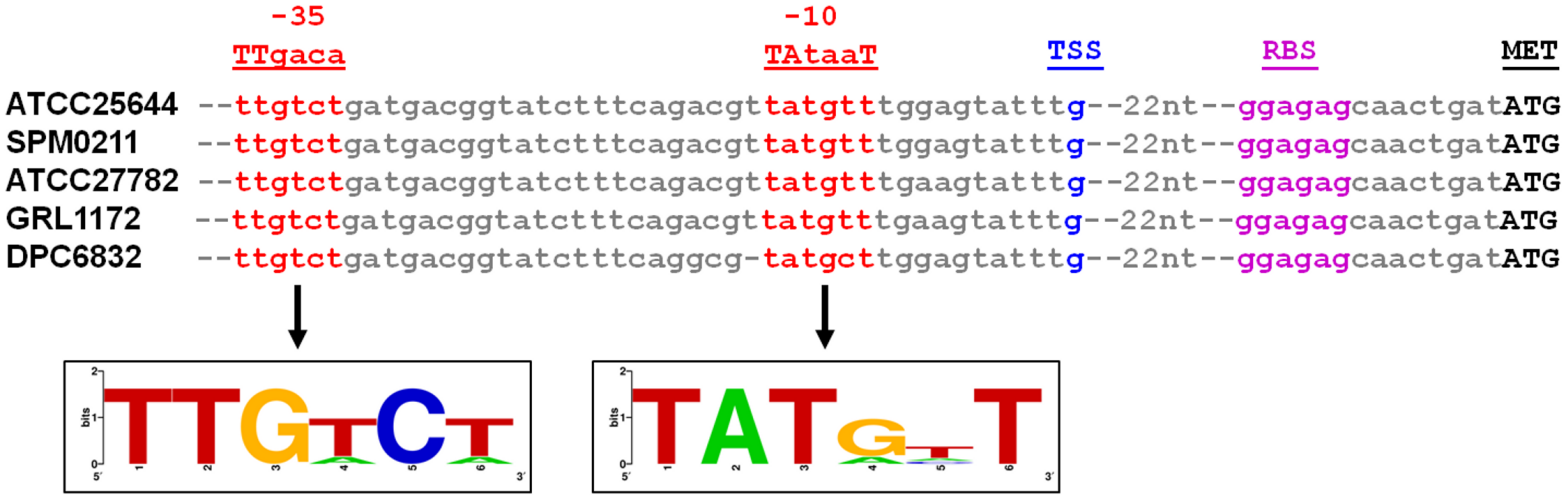
Multiple alignment comparison of the putative promoter sequences for the *L*. *ruminis* fimbrial *LrpCBA* operon. Portions of DNA sequence found directly upstream of the *LrpCBA* pilus operon were retrieved from the genomes of five *L*. *ruminis* strains (ATCC 25644, SPM0211, ATCC 27782, and DPC 6832 via the NCBI database and GRL1172 via our unpublished draft sequencing) and then compiled in a multiple alignment (see further in S3 Fig). Two hexanucleotide elements bearing a resemblance to the canonical -10 (5'-TATAAT-3') and -35 (5'-TTGACA-3') consensus promoter sequences are identified in red font, with the corresponding nucleotides depicted as a WebLogo image [32] (http://weblogo.berkeley.edu/). The putative position for a transcriptional start site (TSS) is indicated in blue font. The length of intervening nt sequence between the promoter region and the possible RBS hexamers (magenta) is specified. The start codon (ATG) of the *lrpC* locus is shown in black boldface type.

As a prior study found from microarray expression profiling of *L*. *ruminis* strains that the *lrpCBA* operon can undergo active transcription [17], we scrutinized the nucleotide region located directly upstream of the *lrpC* locus and sought out any putative promoter sequences capable of regulating pilus-gene expression. For this, we compiled and aligned a 106-nt segment of sequence that was retrieved from a collection of human (ATCC 25644 and SPM0211), bovine (ATCC 27782), equine (DPC 6832), and porcine (GRL1172) host genomes and used it for comparative analysis and evaluation (S3 Fig). This multiple alignment revealed that the nucleotide variability is exceptionally low, with only a few sites having any differences along this sequence length. Of particular pertinence, located adjacently upstream to the presumed ATG (methionine) start codon of the *lrpC* gene, and appearing identical in nucleotide composition among the five aligned DNA segments, there is a recognizably short stretch of sequence (i.e., 5'-GGAGAG-3') for a RBS motif (Fig 2 and S3 Fig). Moreover, within a region further upstream we located a pair of near-optimal spaced six-nucleotide motifs that closely resemble the -10 and -35 consensus promoter elements (i.e., 5'-TATAAT-3' and 5'-TTGACA-3', respectively) necessary for RNA polymerase binding (Fig 2). Here, each of these two putative promoter sequences has four nucleotide positions matching the canonical hexanucleotides. As a purine-type nucleotide (guanine) is well positioned downstream of the proposed -10 element (Fig 2), this base might serve as the starting point for *lrpCBA*-related gene transcription. Taken together, it seems likely that all of these abovementioned nucleotide-motifs constitute the promoter region that controls constitutive expression from the fimbrial *lrpCBA* operon.

### Evidence of native cellular-anchored pilus production in *L*. *ruminis* ATCC 25644

**Fig 3:**
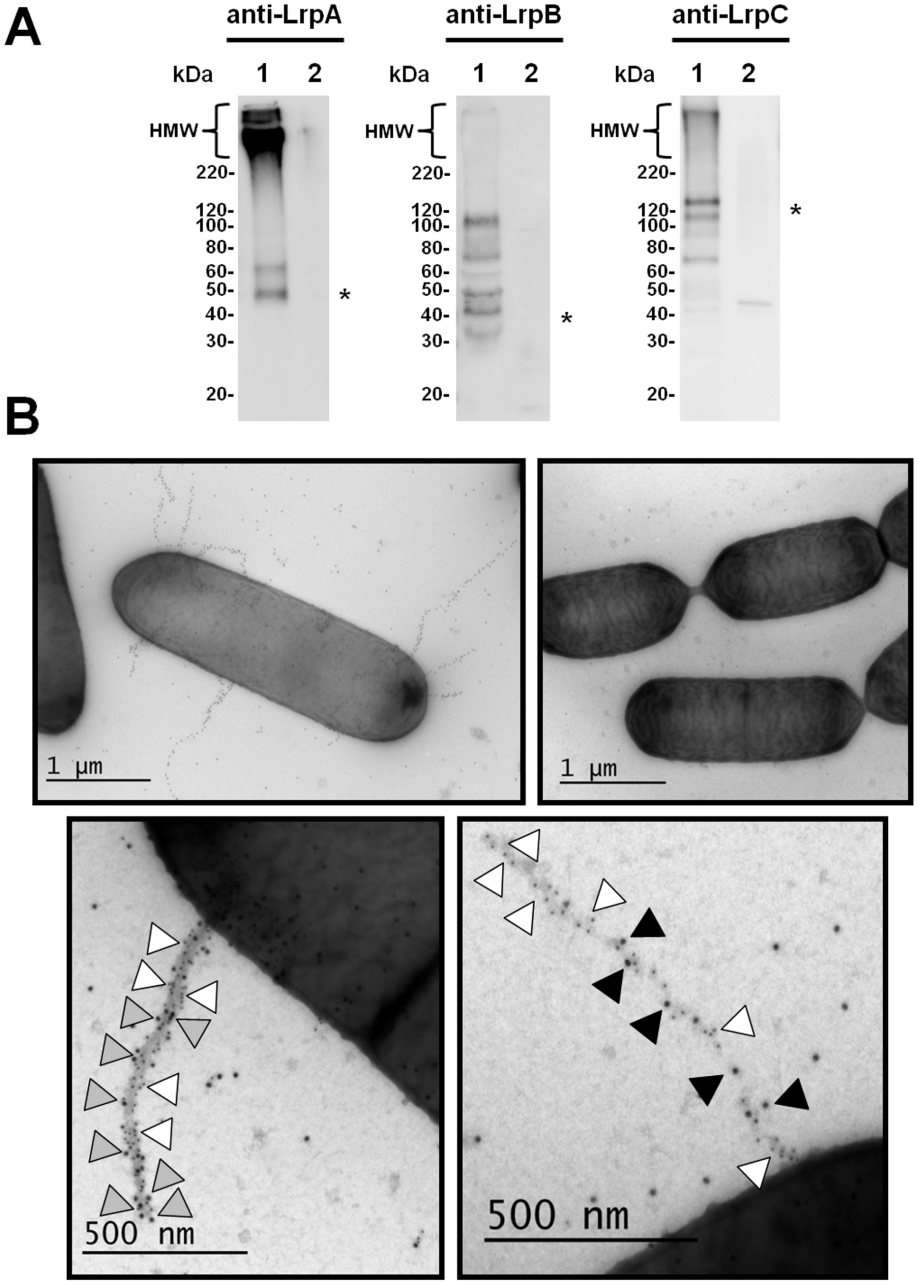
Evidence of *lrpCBA* operon-encoded pilus expression and surface localization in *L*. *ruminis*. **(A)** Immunoblot analysis of LrpCBA piliation in *L*. *ruminis*. Whole-cell preparates of *L*. *ruminis* ATCC 25644 (lane 1) and *L*. *rhamnosus* GG (lane 2) were each immunoblotted and probed with polyclonal anti-LrpA (left panel), anti-LrpB (middle panel), and anti-LrpC (right panel) sera. A condensed laddering of high-molecular-weight (HMW) protein bands, which are indicated along the top left of the immunoblot represents the lengthiest fragments of assembled pili. Protein bands approximating the molecular size of the LrpA (~49 kDa), LrpB (~39 kDa), and LrpC (~123 kDa) monomers are identified by an asterisk on the right side of the immunoblot, whereas the sizing markers for molecular weight (kDa) are shown to the left. **(B)** Immuno-electron microscopic visualization of LrpCBA piliation in *L*. *ruminis*. Immunogold pilin-protein labeling and electron microscopy of *L*. *ruminis* (ATCC 25644) were performed accordingly. *L*. *ruminis* cells were labeled with antiserum against LrpA pilin subunits and protein A-10-nm gold particles (top left panel). The *L*. *rhamnosus* GG strain is used as a control and was treated the same (top right panel). Double labeling of *L*. *ruminis* cells was performed with combined antisera against either LrpA (10-nm; white arrowhead) and LrpC (15-nm; gray arrowhead) pilins (bottom left panel) or LrpA (10-nm; white arrowhead) and LrpB (15-nm; black arrowhead) pilins (bottom right panel). Shown is a representative image from each EM experiment. A scale bar is included at the bottom of each panel.

In determining whether *L*. *ruminis* ATCC 25644 is a surface-piliated strain, we first performed immunoblotting analysis. This represents a tried-and-tested method for establishing sortase-dependent pilus-protein production in Gram-positive bacterial cells. Here, an immuno-detected blotting pattern shows the different sized fragments of assembled pili, which characteristically appears as a laddered length of compacted high-molecular-weight (HMW) protein bands. To obtain the necessary anti-pilin sera for these experiments, we cloned and over-expressed intracellular forms of all three LrpCBA pilin subunits in *Escherichia coli*. These recombinant proteins lacked the pilin-canonical N- and C-terminal signaling and recognition domains, but were instead hexahistidine-tagged at the COOH-terminus to facilitate purification. Purified LrpA, LrpB, and LrpC pilin-proteins were used to raise polyclonal antiserum in rabbits. The corresponding pilin-specific antisera (anti-LrpA, anti-LrpB, and anti-LrpC) were then used to probe membranes that had been blotted with *L*. *ruminis* cells. As clearly evident in the corresponding immunoblots (Fig 3A, lane 1 of all panels), each of the anti-pilin sera gives the distinguishing fingerprint-like band pattern for the presence of assembled pilus structures, which includes the so-termed HMW protein bands, but as well, those protein bands whose apparent sizes are a match to the various LrpCBA pilin monomers. In comparison, a similarly detected blotting pattern was not observed with the *L*. *rhamnosus* GG cells we were using as a negative control (Fig 3A, lane 2 of all panels). From this, we suggest that the active transcription of the fimbrial *lrpCBA* operon in the ATCC 25644 strain observed by others [17] can then be extended to mean the corresponding locus transcripts are in fact also translatable to functional pilin and sortase proteins, with these presumably coming together to yield endogenously formed lengths of LrpCBA pili in *L*. *ruminis* cells.

To reveal more about the multi-subunit structure of the expressed LrpCBA pilus, we next undertook immunogold pilin-protein labeling coupled with electron microscopy (EM) analysis, which for itself continues to be the gold standard of techniques for the cellular visualization of sortase-assembled surface piliation in Gram-positive bacteria. Here, surface-visible pilus formations are identified as the gold particles denoting the position of those types of pilin subunits making up the polymerized pilus. Typically, this is seen in an EM image as a lengthwise collection of black dots, and more than often being outlined by a faint electron-dense zone. For our initial attempt with *L*. *ruminis* ATCC 25644 cells, we conducted single labeling experiments using the antiserum specific for the predicted backbone-pilin subunit (LrpA). Under EM, the cells examined had the visible tell-tale signs of surface piliation, with numerous different-lengthed black-dotted protrusions extending from the cell wall surface (Fig 3B, top left panel). The majority of cells were found to be piliated, with anywhere between one to five pili per cell, but as well, there occasionally being more (or none at all) present. As such, the high number of gold particles associated with the various pilus lengths is consistent with the expected precursor role of the LrpA pilin subunits in assembling into the structural backbone of the LrpCBA pilus. In contrast, and as the negative control, *L*. *rhamnosus* GG cells do not exhibit surface piliation when labeled using the same LrpA antiserum (Fig 3B, top right panel). Thus, our findings here provide clear visual evidence of outward piliation in the *L*. *ruminis* ATCC 25644 strain, but as well, that this surface feature can be construed as structurally dissimilar from the SpaCBA pilus in *L*. *rhamnosus* GG. However, worthy of mention here, as it is normal for a polyclonal mix of anti-pilin antibodies to be used in this sort of EM experiment, it would not be a valid assumption that a one-to-one binding ratio exists between the LrpA antiserum and the corresponding LrpA pilin-protein target. Thus, it is very much conceivable that the various epitopes on a single LrpA pilin-protein subunit could attract the binding of more than one kind of the accessible polyclonal antibody types, which might then be detected as an inflated number of gold particles per individual pilin subunit.

As a means for further visually dissecting the subunit composition of the LrpCBA pilus, we also conducted double labeling EM experiments that include the use of antisera targeting the ancillary LrpB and LrpC pilin-proteins (Fig 3B, bottom panels). Here, for those *L*. *ruminis* cells being immuno-labeled with both the anti-LrpA and anti-LrpC antibodies (Fig 3B, bottom left panel), it is rather obvious from the observed results that the larger sized LrpC pilin is in effect positioned at the pilus tip. However, further interpretation of the gold-particle patterning seems to suggest that this subunit is also dispersed at various sites on the pilus backbone. This latter observation is reminiscent of what had been reported for the *L*. *rhamnosus* GG native SpaCBA [4,5] and recombinant SpaFED [14] pilus structures, and then likewise meaning the added backbone manifestation of LrpC pilins might in fact just represent a possible EM artifact brought on by some antiserum cross-reactivity. Nonetheless, at this point, it is quite reasonable to conclude the *lrpC* locus encodes for the pilin-protein whose main structural role within the LrpCBA pilus is to be deposited and available at the tip location, and as what we surmise is for serving an adhesive function in *L*. *ruminis* cells (for experimental evidence, see sections that follow). When *L*. *ruminis* cells were instead double-labeled with LrpA and LrpB antisera, the gold-particle protrusions extending outward are mainly comprised of those for the LrpA pilin-proteins, but interspersed throughout as well are a few for the predicted basal LrpB pilin subunit (Fig 3B, bottom right panel). According to the corynebacterial pilus-assembly model [33], basal pilins that occur along the Gram-positive pilus structure are presumed to be a random event, rather possibly being a reflection of subunit misincorporation into the growing lengths of pili. However, regarding the established consensus view of base-situated pilins and their foremost location, LrpB is likely to conform similarly, and in spite of being concealed within the cell wall structure and going visibly unnoticed during our EM examinations. In summary, it should be noted that with the results described in this section for *L*. *ruminis* ATCC 25644, this now represents just the second reported example of a native-piliated *Lactobacillus* species, but additionally, where a third lactobacillar pilus type has been revealed and branded by immuno-EM.

### Recombinant cellular expression of the fimbrial *lrpCBA* operon in *L*. *lactis*

To identify and characterize some of the phenotypic properties of the *L*. *ruminis* pilus, we genetically engineered the fimbrial *lrpCBA* operon into *L*. *lactis* for the nisin-induced recombinant production of cell surface-assembled wild-type (WT) LrpCBA pili. This was done as an alternative to constructing a non-piliated *L*. *ruminis* mutant strain. Previously, we had used the same cloning tactic in our studies of the *L*. *rhamnosus* GG *spaCBA* and *spaFED* operon-encoded pili, and where *Lactococcus* cells with these forms of piliation were proven reliably quite effective for acquiring phenotypically pertinent data and information [11,14]. In fact, some successful examples of using this cloning approach with pilus types from other Gram-positive bacteria are also well documented [34–38].

**Fig 4:**
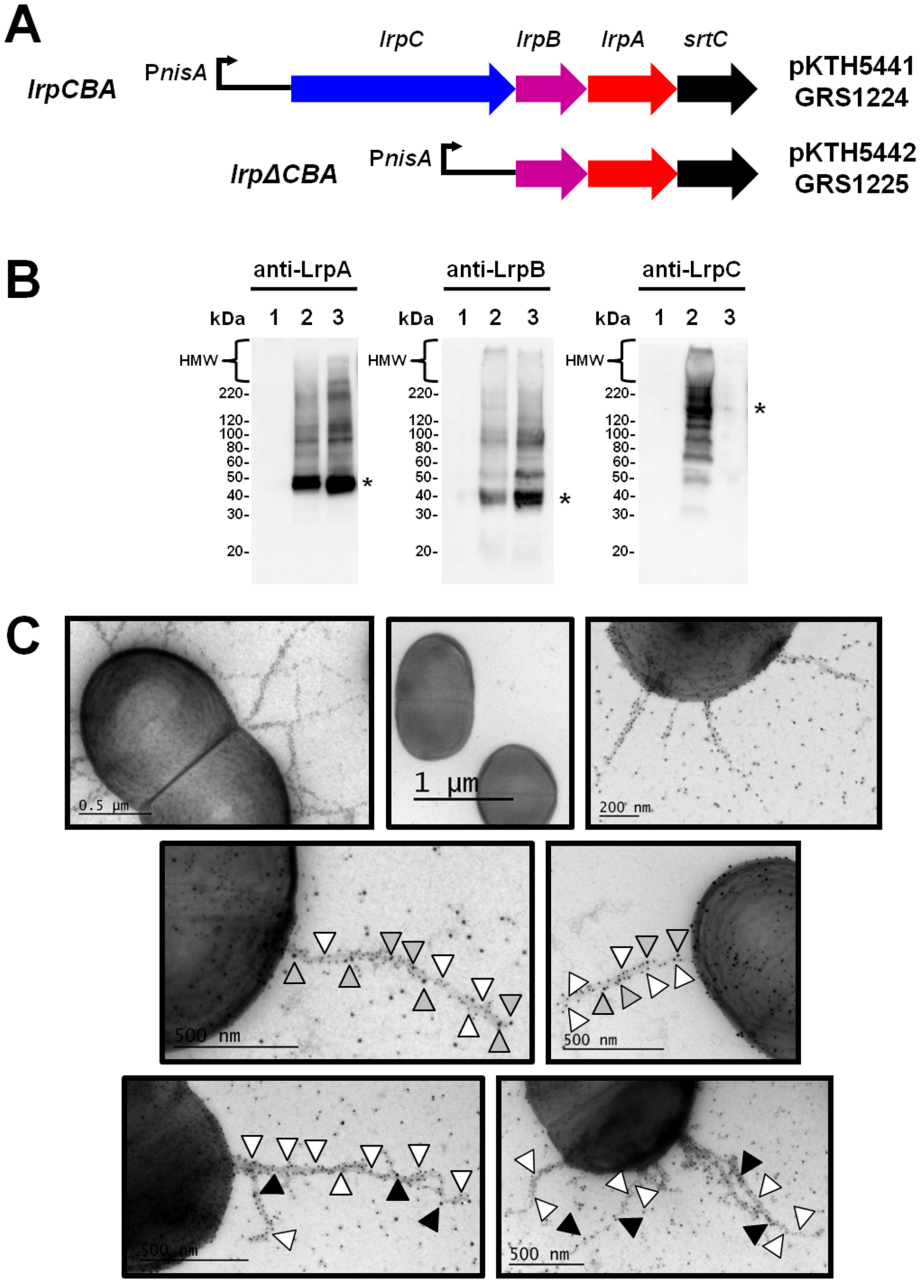
Recombinant surface expression of *L*. *ruminis* LrpCBA pili on lactococcal cells. **(A)** Schematic representation of the gene constructs used for generating the WT and LrpC-deleted LrpCBA-piliated lactococcal clones. For the recombinant WT lactococcal plasmid construct (pKTH5441), the four genes (*lrpC*, *lrpB*, *lrpA*, and *srtC*) in the fimbrial *lrpCBA* operon were cloned under the control of the nisin-inducible *nisA* promoter (P*nisA*). The LrpC-deleted plasmid construct (pKTH5442) was cloned in the same way, but with the *lrpC* locus removed from the coding region of the *lrpCBA* operon. The WT and Δ*lrpC* mutant-bearing plasmids are each propagated in *L*. *lactis* NZ9000 to produce two recombinant clones (GRS1224 and GRS1225, respectively). **(B)** Immunoblot analysis of recombinant LrpCBA pili produced in *L lactis*. Immunoblots of nisin-induced cultures of the GRS1052 (lane 1), GRS1224 (lane 2), and GRS1225 (lane 3) recombinant lactococcal clones were prepared and then treated with polyclonal antisera against LrpA (left panel), LrpB (middle panel), or LrpC (right panel) pilin subunits. High-molecular-weight (HMW) protein bands representing the longer assembled pilus fragments are identified near the top of each immunoblot and marked accordingly to the left side. An asterisk placed on the right side of the immunoblot approximates the position and size of monomeric pilin-protein (i.e., ~49-kDa LrpA, ~39-kDa LrpB, and ~123-kDa LrpC). Markers for molecular size (kDa) are indicated on the left. **(C)** Immuno-electron microscopic detection of cell surface-localized recombinant LrpCBA pili in *L lactis*. Immunogold pilin-protein labeling and electron microscopy of the GRS1224 (WT) and GRS1225 (Δ*lrpC*) recombinant LrpCBA-piliated lactococcal clones as well as the vectorless GRS71 strain were done accordingly. GRS1224 (top left panel) and GRS1225 (top right panel) cells were immuno-labeled using anti-LrpA serum and protein A-10-nm gold particles. GRS71 cells served as a control and were treated in a likewise manner (top middle panel). Double labeling experiments were carried out for GRS1224 (middle left panel) and GRS1225 (middle right panel) cells with an antisera mix targeting LrpA (10-nm; white arrowhead) and LrpC (15-nm; gray arrowhead) pilins. GRS1224 (bottom left panel) and GRS1225 (bottom right panel) cells were as well double-labeled with antisera specific for LrpA (10-nm; white arrowhead) and LrpB (15-nm; black arrowhead) pilins. Representative EM images are chosen and shown. A scale bar is included in each panel.

Thus, to generate recombinant WT LrpCBA-piliated lactococci (denoted GRS1224), we used a nisin-controlled expression vector (pKTH5080; unpublished) for cloning the coding regions of the four loci (*lrpC*, *lrpB*, *lrpA*, and *srtC*) of the *L*. *ruminis* ATCC 25644 *lrpCBA* pilus operon. The resultant plasmid, named pKTH5441 and now having a nisin-inducible fimbrial *lrpCBA* operon, was transformed into the *L*. *lactis* NZ9000 strain. To probe further the functionality of the LrpCBA pilus and target specifically the putative adhesive role of the tip-positioned LrpC subunit (e.g., its predicted collagen-binding trait), we made an additional LrpCBA-piliated lactococcal clone (GRS1225) that was unable to produce the LrpC pilin-protein. For this, the *lrpC* locus was deleted from the coding region of the *lrpCBA* pilus operon, with the Δ*lrpC* mutant-bearing plasmid (called pKTH5442) also propagated in the NZ9000 strain. The gene arrangement of both recombinant constructs is schematically provided in Fig 4A. To evaluate the authenticity of LrpCBA pilus production in each of the GRS1224 and GRS1225 lactococcal clones, we analyzed nisin-induced cells first by immunoblotting, and then by immuno-EM. For these experiments, we utilized the same three different LrpCBA pilin antisera that had been used previously to demonstrate native LrpCBA piliation in *L*. *ruminis* ATCC 25644 (see preceding section).

As shown in Fig 4B, immunoblotting analysis of the GRS1224 (lane 2) and GRS1225 (lane 3) recombinant constructs with the antiserum specific for the LrpA backbone-pilin subunit (left panel) suggests each of the lactococcal clones is likely piliated, as evidenced by the immuno-detection of the pilus-associated HMW protein bands. Moreover, when anti-LrpB (middle panel) and anti-LrpC (right panel) sera were used for immunoblotting, the blotted patterns seen in lanes 2 and 3 suggest the existence of the corresponding ancillary pilin-proteins in the recombinant pilus structures of each lactococcal clone, but with the exception of GRS1225, where the absence of any LrpC tip-pilin production is so confirmed (right panel, lane 3). As a negative control, whole-cells of the non-piliated empty vector GRS1052 clone were blotted and probed with each of the three LrpCBA pilin-specific antisera (all panels, lane 1).

The above-mentioned immunoblotting data were further substantiated visually by performing a set of EM experiments that involved both single and double immunogold labeling of the LrpA, LrpB, and LrpC pilin-proteins (Fig 4C). For instance, when nisin-treated GRS1224 (top left panel) and GRS1225 (top right panel) whole-cells were single-labeled with anti-LrpA serum, it was apparent from the EM images that each clone is visibly showing surface piliation, as seen extending outward from the cells are the recognizable patterned lengths of pilin-detected gold particles (black dots). As a negative control, when the above EM experiment was done with the recombinant host strain (GRS71, i.e., *L*. *lactis* NZ9000), there is no comparable indication of any surface-localized pilus formations (top middle panel). Added to this, our double labeling experiments carried out with both recombinant lactococcal clones using pilin-antisera mixes that target either the LrpA and LrpC subunits (middle left panel for GRS1224 and middle right panel for GRS1225) or the LrpA and LrpB subunits (bottom left panel for GRS1224 and bottom right panel for GRS1225) support the immunoblot findings.

It should be mentioned that with some of the EM images (Fig 4C) the gold particle background seemed somewhat high, but since negligible background was seen with the various preimmune serum controls (S4 Fig), we ruled out non-specific antibody binding as the reason behind these additional gold particles. Instead, we took their presence to be from the extra amount of broken pilus fragments that are associated with the lactococcal clones. As the recombinant pili tended to be longer it seemed that they were prone to breakage once exceeding a certain length, with these remnants then proving to be difficult to rinse away completely during immunogold labeling. Moreover, concerning the Δ*lrpC* mutant construct (GRS1225), although the corresponding pili are visually shown to be missing the LrpC subunit at the tip location, there was some tendency for related gold particles to be still observed along the backbone, this despite rigorous washing of the EM grids. Because a similar phenomenon was not detected for the corresponding immunoblot (see preceding section), we instead reason this as an artifact that perhaps originates from polyclonal cross-reactivity with LrpA subunits, but yet that inexplicably only occurs when immunogold labeling is being performed, and then not during immunoblotting experiments.

As seen in our earlier work [11,14], it appears that nisin-induced pilus expression in the two LrpCBA-piliated clones is not overly uniform, this then noticed as the fluctuated numbers of pili per cell, which on average would range between zero and five. However, much more relevantly, for any subsequent phenotypic characterizations of *L*. *ruminis* piliation using the GRS1224 and GRS1225 lactococci (see sections that follow), and as inferred from our EM-interpreted observations, it can be safely argued that the native- and recombinant-produced LrpCBA pilus forms bear a striking resemblance at the gross structural level. Consequently, with there being a perceived authenticity for the recombinant-produced LrpCBA pilus, this strengthens the feasibility and use of the two corresponding clones for functional studies on the surface piliation seen with *L*. *ruminis* ATCC 25644.

### Certain niche-desirable binding functionalities are attributable to LrpCBA piliation

Based on accumulated data over the years [1,8,14], it would seem that most types of Gram-positive pili share a common functional predisposition for adherence to extracellular matrix (ECM) proteins. As described previously (see earlier section above), our Pfam searches revealed that the primary structure of the ancillary (and tip-localized) LrpC pilin subunit contains a putative domain for binding to collagen (S2 Fig), and with that, which could then be expected to engage in some aspect of *L*. *ruminis* cellular adhesion to this particular ECM component. Accordingly, it was our intention to test this premise out by using the recombinant-piliated GRS1224 and GRS1225 constructs and measure their *in vitro* binding capacities for collagen, but as well, for certain other substrates.

#### Collagen binding

**Fig 5:**
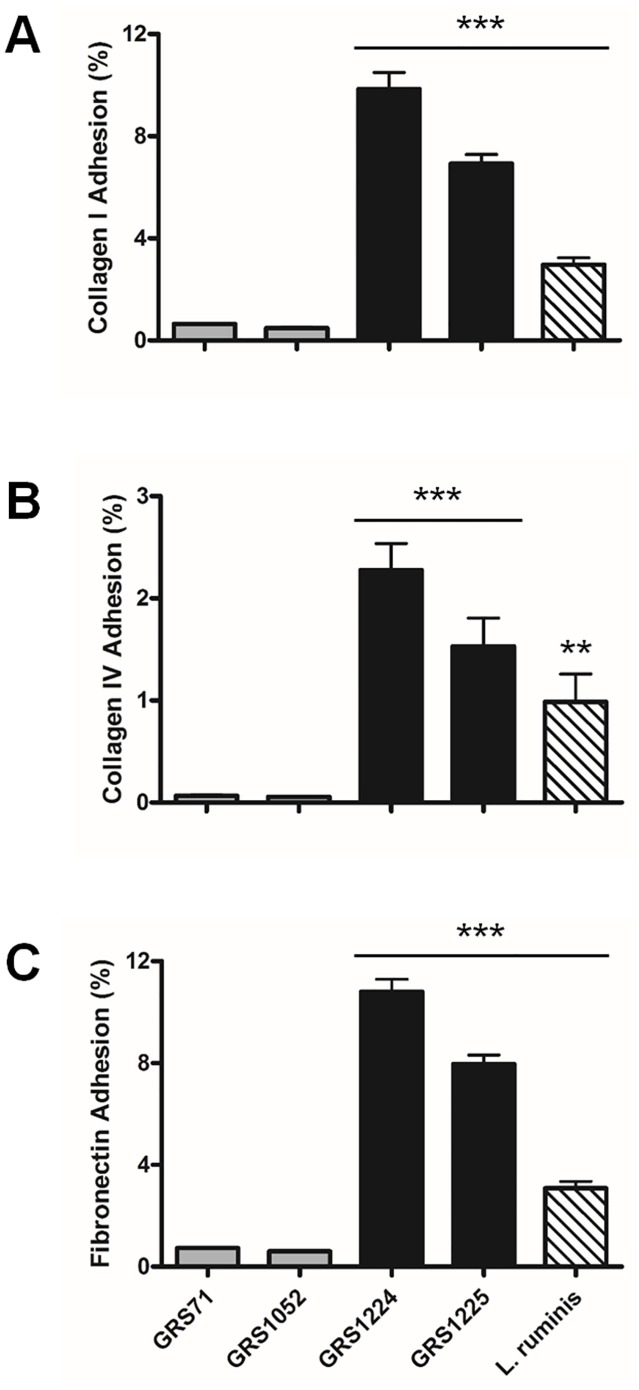
LrpCBA pilus-mediated cellular adhesion to ECM-related proteins. Normalized (OD600 = 0.5) cultures of *L*. *ruminis* cells and the recombinant WT and LrpC-deleted LrpCBA-piliated lactococci (GRS1224 and GRS1225, respectively) were assayed for *in vitro* binding to collagen type I **(A)**, type IV **(B)**, and fibronectin **(C)**. Vectorless (GRS71) and empty vector (GRS1052) lactococci were included as controls in these experiments. Measurements were normally made in triplicate, with the standard error of mean (SEM) shown by limit bars. Statistical differences for individual pairwise comparisons against the GRS71 control are denoted as *** = *P* ≤ 0.001 (highly significant) or ** = *P* ≤ 0.01 (very significant).

Importantly, and as seen from Fig 5A and 5B, it is first clear that *L*. *ruminis* ATCC 25644 is itself able to adhere to collagen protein, and at levels that are much higher with type I than type IV. Thus, we can imagine that among the many possible surface-exposed adhesins of *L*. *ruminis*, this sort of ECM-binding activity might at least be partly associated with the predicted collagen adhesiveness of the LrpCBA pilus. In fact, when nisin-induced WT LrpCBA-piliated GRS1224 cells were measured for binding to collagen, a conspicuously high level is detected for type I (Fig 5A). Conversely, adherence by the GRS1224 clone to collagen type IV is far less pronounced (Fig 5B), but still appreciably more than detected with the negative control cells (GRS71 and GRS1052). From these results, it now seems quite certain that *L*. *ruminis* LrpCBA piliation not only has a substrate-binding specificity substantiating prior Pfam domain predictions (S2 Fig), but as well, that this lactobacillar pilus is in step with other Gram-positive pili by exhibiting a measurable affinity for collagen. Moreover, the levels of collagen-binding activity for the LrpC-deleted GRS1225 clone exhibit a reduction (albeit not entirely), and then more so for type I than type IV (Fig 5A and 5B). Taken from this, it seems that the LrpC pilin subunit has a needed binding role in the collagen-targeted functionality of LrpCBA piliation.

#### Fibronectin binding

Because some Gram-positive pili also have a proven binding affinity for fibronectin, itself another of the ECM proteins, we examined *L*. *ruminis* surface piliation to see whether it behaves likewise and shares the same substrate specificity. Previously, we showed that the *L*. *rhamnosus* GG SpaFED pilus has a binding affinity that is multi-targeted and includes adherence to fibronectin protein [14]. For our present study, we found that the *L*. *ruminis* ATCC 25644 strain has its own inherent adhesiveness for this particular ECM component. From data shown in Fig 5C, it is recognizable that *L*. *ruminis* cells can bind to fibronectin, and that this measured level of adhesion is in a range slightly higher than found with collagen protein. While it can presumed that such adherence to fibronectin might be multi-factorial, with a host of surface proteins having a conceivable role, we tested if the LrpCBA pilus is also having some sort of functional involvement. This would be despite the fact that none of the three pilin subunits shares homology with any of the known fibronectin-binding domains in the Pfam database. Yet, even so, when WT LrpCBA-piliated GRS1224 cells were analyzed for binding affinity (Fig 5C), we found they are also rather adherent to fibronectin, and clearly more so than the negative control GRS71 and GRS1052 cells. In addition, based on our assessment of recombinant GRS1225 lactococci we suggest that the focal nature of this binding activity is the LrpC subunit, as the LrpC-deleted clone has a diminished ability to adhere fibronectin (Fig 5C).

#### Mucus adhesion

In contrast to the mucus-binding affinity unveiled previously for the lactobacillar SpaCBA and SpaFED pili [4,6,11,14], our evidence of a similar functionality for *L*. *ruminis* LrpCBA piliation was inconclusive. Here, the measured mucus adhesion for the LrpCBA-piliated GRS1224 and GRS1225 clones could only be construed as being of a non-specific artifactual nature (data not shown). This is somewhat expected as it not only seems that the *L*. *ruminis* genome (ATCC 25644) does not encode for any proteins (or pilins) having recognizable mucus-binding domains, but also from our adhesion experiments with the ATCC 25644 strain (data not shown), *L*. *ruminis* can be regarded as a weak and ineffectual binder of mucus. Thus, it is little surprise that LrpCBA piliation might not possess mucoadhesive properties of any functional relevance.

#### Adherence to intestinal cells

**Fig 6:**
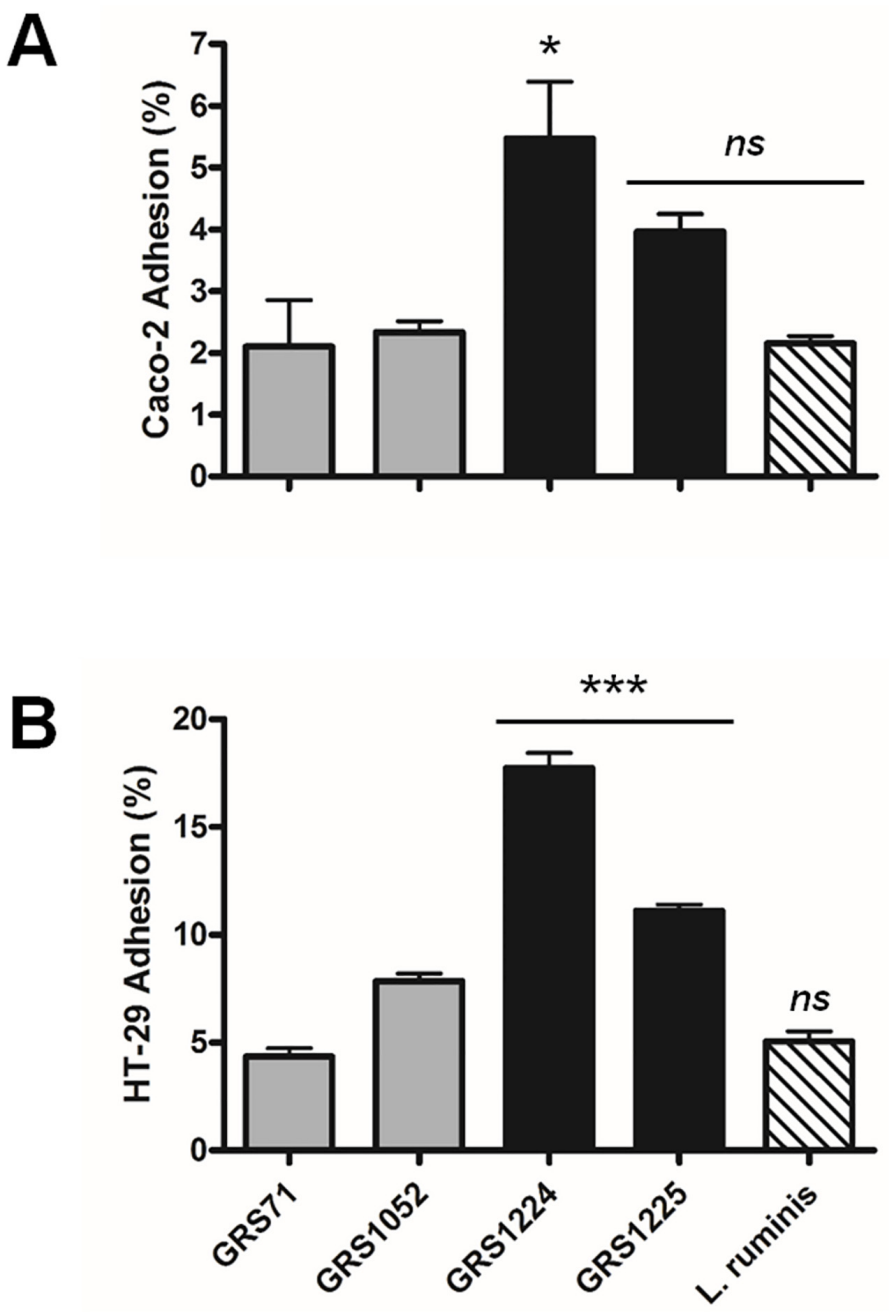
LrpCBA pilus-mediated cellular adhesion to gut epithelial cells. Assessment of LrpCBA pilus-mediated cell-to-cell adhesion between normalized (OD600 = 0.5) cultures of *L*. *ruminis*, and as well recombinant GRS1224 (WT) and GRS1225 (LrpC-deleted) lactococci, and each of the human intestinal Caco-2 **(A)** and HT-29 **(B)** cell lines was performed. GRS71 and GRS1052 cells were included as controls. Measurements were performed in triplicate for both sets of experiments. Limit bars indicate the SEM. Statistical differences for individual pairwise comparisons against the GRS71 control are designated as *** = *P* ≤ 0.001 (highly significant), * = *P* ≤ 0.05 (significant), or *ns* = *P* > 0.05 (not significant).

To get some indication of the functional role for LrpCBA piliation during bacteria-host cell interactions, we assessed whether the human Caco-2 and HT-29 gut epithelial cell lines can act as binding targets for *L*. *ruminis* (ATCC 25644) and recombinant LrpCBA-piliated lactococcal cells (GRS1224 and GRS1225). In this regard, measurable adherence between either *L*. *ruminis* or GRS1224 cells and each of the two intestinal cell lines is noticeably detected (Fig 6A and 6B), and at levels well above the intrinsic binding of the negative control cells (GRS71 and GRS1052). Still, it is worth mentioning that there is some discrepancy in the detected levels of epithelial cell adherence between *L*. *ruminis* and GRS1224 cells, and of the two with *L*. *ruminis* showing lesser binding. Because the cellular LrpCBA pilus production is either constitutive (*L*. *ruminis*) or the result of nisin-induction (GRS1224), we impute the variance in binding ability to the differing numbers of pili per each cell type. More pertinently, and also consistent with results already mentioned (see above), since a lowered adherence to the Caco-2 and HT-29 cells is measured for the GRS1225 clone (Fig 6A and 6B), this again corroborates the LrpC subunit as a binding determinant of the LrpCBA pilus.

#### Biofilm formation

**Fig 7:**
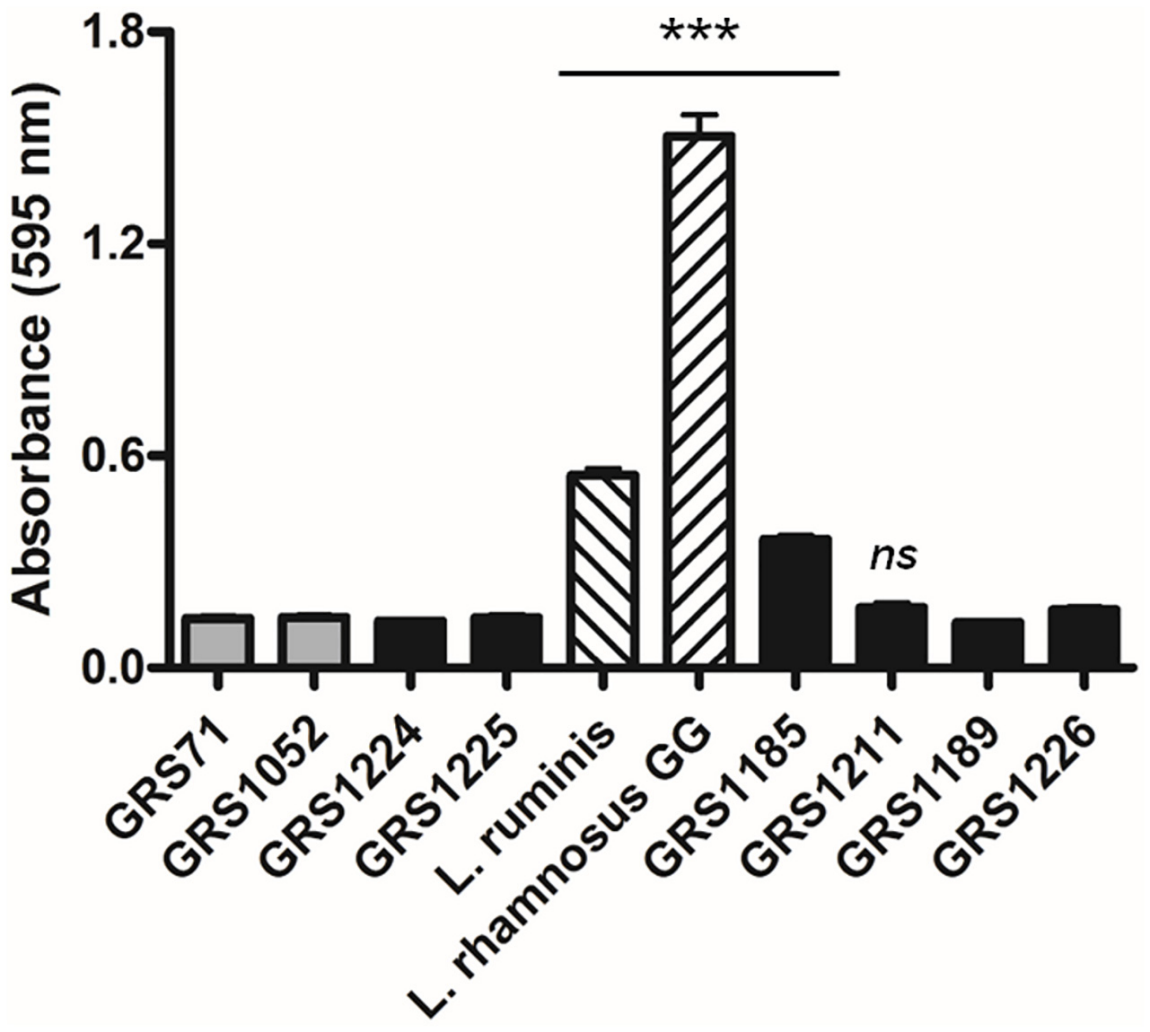
LrpCBA pilus-mediated effects on cellular biofilm formation. Normalized cultures of *L*. *ruminis* cells and the recombinant GRS1224 (WT) and GRS1225 (LrpC-deleted) lactococci were assessed for static biofilm formation using a crystal violet staining assay. Various cellular controls were used and treated in the same manner. These included the pilus-less GRS71 and GRS1052 lactococci, the recombinant WT and SpaC-deleted SpaCBA-piliated (GRS1185 and GRS1211, respectively) and WT and SpaF-deleted SpaFED-piliated (GRS1189 and GRS1226, respectively) lactococcal clones, and the SpaCBA-piliated *L*. *rhamnosus* GG strain. Multiple measurements were taken and an average value calculated (*n* = 7). Limit bars show the SEM. Statistical differences for individual pairwise comparisons against the GRS71 control are indicated as *** = *P* ≤ 0.001 (highly significant) or *ns* = *P* > 0.05 (not significant).

Given that the surface piliation found in various Gram-positive genera and species (for review, see [39,40]), including *L*. *rhamnosus* GG [9], has been implicated in the ability of these bacteria to self-aggregate into biofilm communities, we pursued whether a similar phenotypic trait holds for the *L*. *ruminis* LrpCBA pilus as well. According to our *in vitro* measurements (Fig 7), indications of biofilm development were readily perceived for *L*. *ruminis* ATCC 25644, and likewise for the *L*. *rhamnosus* GG strain, which served as one of the positive controls. Previously, it was concluded from knockout phenotypes that the SpaCBA pilus represents one of the adhesive mechanisms through which biofilm formation in *L*. *rhamnosus* GG presumably can occur [9]. For our present biofilm experiment we also used the cloned SpaCBA-piliated GRS1185 (WT) and GRS1211 (SpaC-deleted) lactococcal constructs [11] as more specific positive controls (Fig 7). With the GRS1185 clone, SpaCBA piliation is shown to participate in forming biofilm, with the SpaC subunit seemingly to have a focal role during the accompanying aggregation interactions (as deduced from the GRS1211 clone). Parenthetically, we noted that this activity is not shared by *L*. *rhamnosus* GG SpaFED pili, as the WT-piliated GRS1189 and SpaF-deleted GRS1226 lactococcal clones [14] do not contribute to biofilm assembly (Fig 7). Nonetheless, it is still rather surprising that when the WT LrpCBA-piliated GRS1224 clone was examined, the capacity for producing biofilm is not at all obvious, appearing nearly the same as the negative control cells (GRS71 and GRS1052) (Fig 7). This suggests the biofilm-forming phenotype in *L*. *ruminis* is unrelated to the ECM-binding functionality of the LrpCBA pilus. In terms of host colonization strategies, this clearly sets *L*. *ruminis* surface piliation apart from *L*. *rhamnosus* GG SpaCBA pili and other Gram-positive pilus types. Evidently, the need for gut-dwelling *L*. *ruminis* to have cell-surface pili geared toward biofilm development has not emerged as a particularly significant evolutionary advantage during niche adaptation and survival.

In the general ecology of *L*. *ruminis* and its apparent autochthonous behavior, having a bi-functional binding specificity that involves adherence to collagen and fibronectin substrates makes the LrpCBA pilus rather effective for promoting cellular adhesion to a gut epithelium surface, particularly to those regions where the concealed ECM components lying buried beneath have become uncovered and more open to bacterial attachment. Moreover, as a species with some proclivity for motility, but not having any strong mucoadhesiveness, *L*. *ruminis* will then be much more adept at maneuvering through the mucus-layer barrier to reach this epithelial cell lining. In addition, given that *L*. *ruminis* cells can undergo anaerobic respiration, they are seemingly well suited for successfully establishing a stable population within the oxygen-poor confines of a gut-niche locale. As such, the adhesive and first-contact nature of LrpCBA piliation can be recognized as one of the adaptive strategies that helps ensure cellular survival within this intestinal microcosm, where host colonization by *L*. *ruminis* then becomes sustainable and presumably lifelong.

### Resolving some of the molecular immunogenic characteristics of LrpCBA piliation

In response to the indigenous commensals comprising the microbiota of the gut, host-immune cells are presumed to initiate basal defense mechanisms that then regulate and maintain a homeostatic tolerance to such bacteria [41–43]. Purportedly, the overall extent of these immunomodulatory activities is characteristically much reduced, if not rather subdued, when compared with the generally heightened level of intestinal immune responsiveness toward an invading and proliferating population of pathogens [41–43]. Yet, common to both situations is the specific interplay between a series of different microbe-associated molecular patterns (MAMPs) on the bacterial cell surface and an assortment of pattern recognition receptors (PRRs) (e.g., the Toll-like receptors; TLRs) expressed by certain types of host-immune cells [41–43]. Concerning the sortase-assembled pilus, it represents a prominent macromolecular cell-surface structure that has been correlated with an immunostimulating capacity, as in *Streptococcus pneumoniae* [44] and *Bifidobacterium bifidum* [45]. Previously, we showed that the *L*. *rhamnosus* GG SpaCBA pilus could induce TLR2-dependent activation in immune-related cells and modulate human monocyte-derived dendritic cell (moDC) cytokine production [11]. Interestingly, for both these immuno-activities the adhesiveness of the SpaC pilin subunit is seen to have some causal influence. In fact, the adhesive functionality of SpaC has been linked both directly and indirectly to several other immunomodulating actions [9,10,12,13]. However, in a more recent study of ours, we found unexpectedly that the *L*. *rhamnosus* GG SpaFED pilus instead causes a dampening of the induced immune responses being roused in certain intestinal- and immune-related cell types [14]. With these various effects in mind, we undertook an attempt to resolve the innate immunostimulatory potential of *L*. *ruminis* LrpCBA piliation.

**Fig 8:**
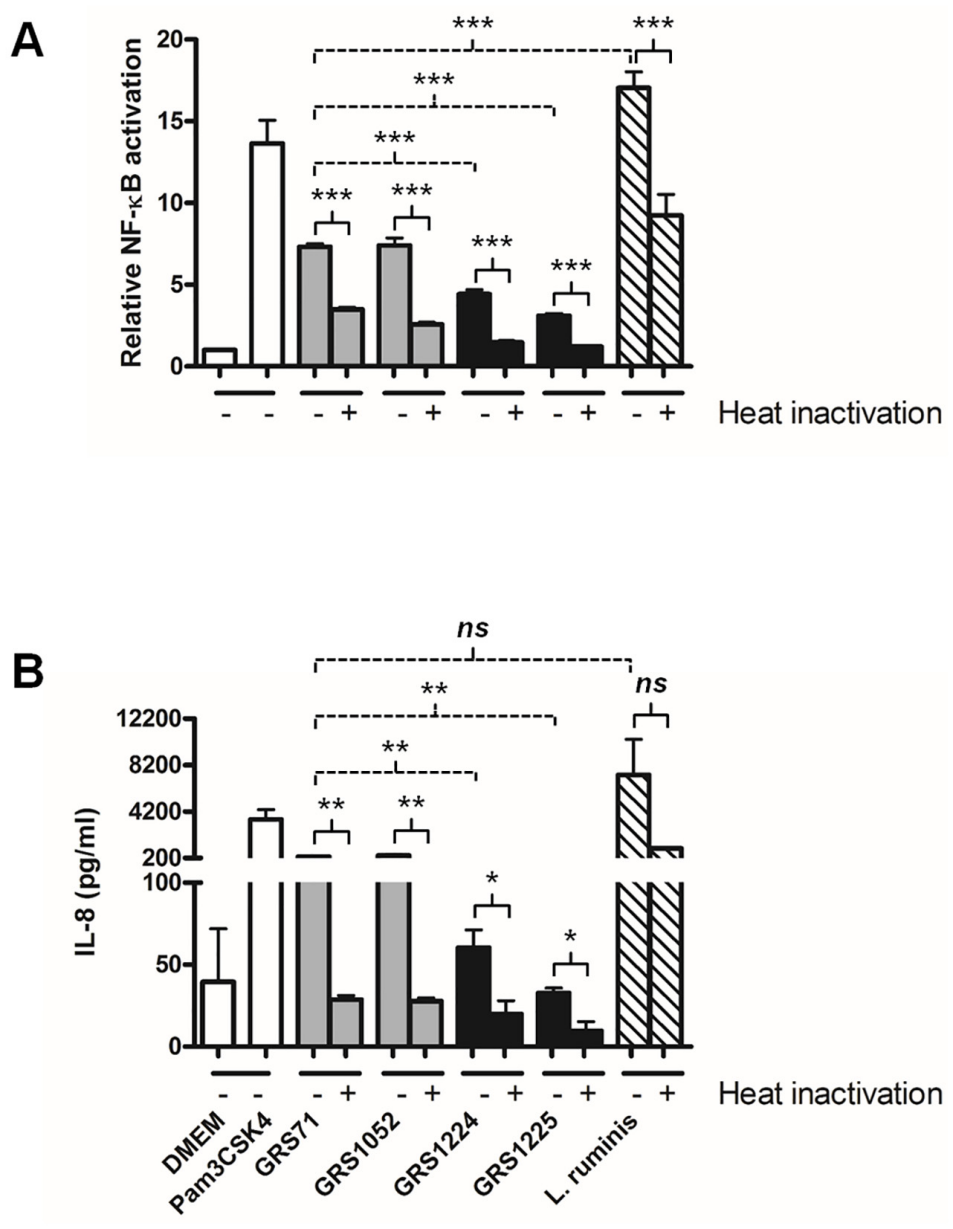
LrpCBA pilus-mediated cellular variation of TLR2-regulated NF-κB responses and endogenous IL-8 production in live and heat-treated HEK-TLR2 cells. Live (-) and heat-treated (+) normalized cultures of *L*. *ruminis* cells and the recombinant GRS1224 (WT) and GRS1225 (LrpC-deleted) lactococci (MOI 100) were combined with HEK-TLR2 cells. TLR2-dependent NF-κB activation **(A)** and endogenous IL-8 production **(B)** were measured. GRS71 and GRS1052 cells were used as controls and treated similarly. Measurements also taken for DMEM cell-culture medium and a TLR2-agonist lipopeptide (Pam3CSK4; 1 ng/ml) were used as negative and positive controls, respectively. Triplicate measurements were made for both sets of experiments. Limit bars show SEM. Statistical differences for individual pairwise comparisons against the GRS71 control (unheated) are specified as *** = *P* ≤ 0.001 (highly significant), ** = *P* ≤ 0.01 (very significant), or *ns* = *P* > 0.05 (not significant). Individual pairwise comparisons of data between the heated and unheated samples are indicated as *** = *P* ≤ 0.001 (highly significant), ** = *P* ≤ 0.01 (very significant), * = *P* ≤ 0.05 (significant), or *ns* = *P* > 0.05 (not significant).

**Fig 9:**
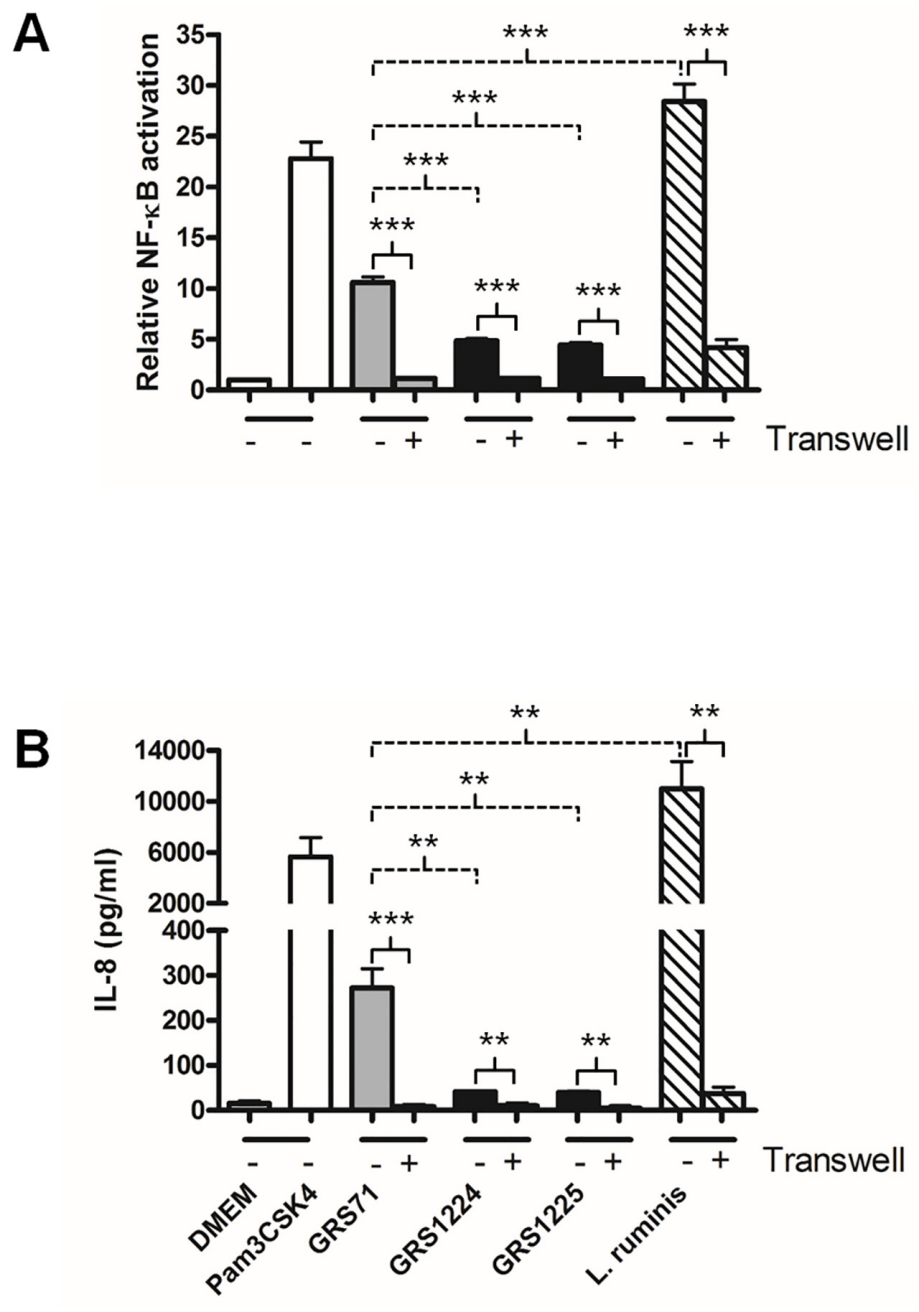
Influence of cell-to-cell interactions on the LrpCBA pilus-mediated cellular variation of TLR2-regulated NF-κB responses and endogenous IL-8 production in HEK-TLR2 cells. Normalized cultures of *L*. *ruminis* cells and the recombinant GRS1224 (WT) and GRS1225 (LrpC-deleted) lactococci (MOI 100), either non-partitioned (-) or Transwell-partitioned (+), were added to HEK-TLR2 cells, with the levels of TLR2-dependent NF-κB activation **(A)** and endogenous IL-8 production **(B)** then quantified. GRS71 cells (MOI 100), DMEM cell-culture medium, and Pam3CSK4 (1 ng/ml) were also included as controls. Measurements for both sets of experiments were done in triplicate, with limit bars representing the SEM. Statistical differences for individual pairwise comparisons against the GRS71 control (without Transwell membranes) as well as the individual pairwise data comparisons of samples with or without Transwell membranes are denoted as *** = *P* ≤ 0.001 (highly significant) or ** = *P* ≤ 0.01 (very significant).

To determine whether the LrpCBA pilus evokes any TLR2 agonist-like responses, we used a commercially available recombinant HEK293 cell line (HEK-TLR2) that has been modified to contain the human TLR2 gene and the extra loci for a NF-κB (nuclear factor-kappa beta)-based secretable alkaline phosphatase (SEAP) reporter system. For this, we measured the extent to which *L*. *ruminis* and the GRS1224 and GRS1225 recombinant clones are able to induce any fluctuative change to the TLR2 signaling profile and the levels of extracellularly secreted IL-8. As shown by Fig 8A and 8B, *L*. *ruminis* (ATCC 25644) can induce rather conspicuously elevated levels of both TLR2-dependent NF-κB activation and endogenous IL-8 production. However, since these levels become only partly reduced in experiments where *L*. *ruminis* cells had been either exposed to a protein-denaturing temperature (Fig 8A and 8B) or partitioned by Transwell cell culture membranes (Fig 9A and 9B), this suggests that just a portion of these two types of immunostimulating activities can be accounted for by proteins that are heat susceptible and attached to the cell surface. While the remaining recalcitrant fraction of elicited immuno-responsiveness might possibly derive from a speculatively less heat-labile LrpCBA pilus, it is more likely due to other types of proteinaceous and carbohydrous entities that are of a combined cell-released, surface-attached, or possibly heat-resistant nature. Nonetheless, in this context it is still quite surprising that the WT-piliated GRS1224 and LrpC-deleted GRS1225 clones exhibit stimulated TLR2-related activity and IL-8 secretion at levels that are appreciably much less than detectable for the negative control cells (GRS71 and GRS1052) (Fig 8A and 8B and Fig 9A and 9B). As an interpretation, we equate the *L*. *ruminis* LrpCBA pilus with the reported behavior of *L*. *rhamnosus* GG SpaFED piliation [14], where it also serves to dampen the *Lactococcus*-induced immuno-responsiveness of the HEK-TLR2 cell line, but without the ECM-adhesive LrpC subunit having a causal role. Moreover, it is of some noteworthiness that following the high temperature and Transwell treatments of the GRS1224, GRS1225, GRS1052, and GRS71 cells, for each of them the induced immuno-responsiveness from the HEK-TLR2 cells had dropped near the basal level seen with the DMEM medium control (Fig 8A and 8B and Fig 9A and 9B). Such results lead us to speculate that only surface proteins are likely to be involved, and perhaps are then responsible for stimulating the so-tested immunomodulations.

**Fig 10:**
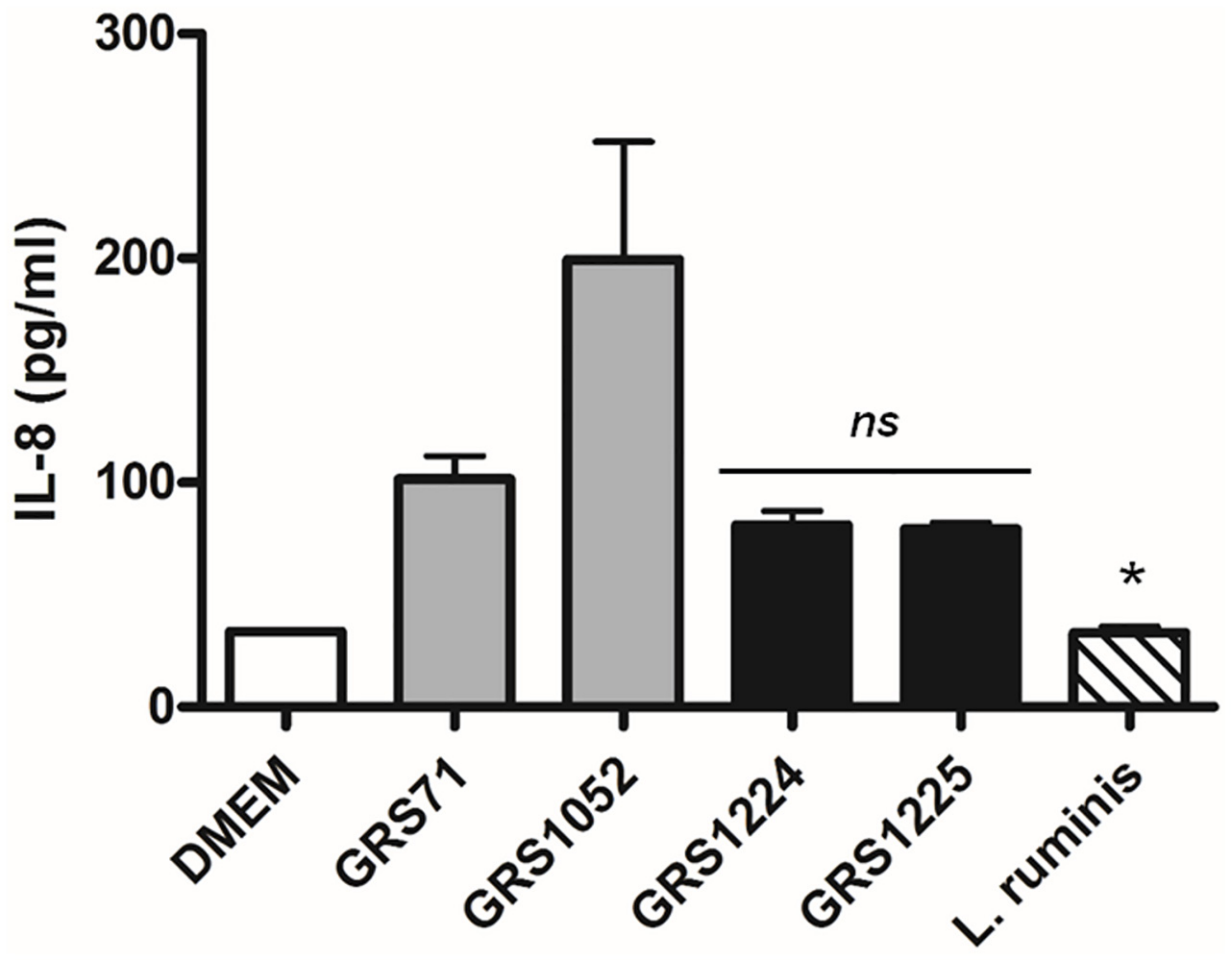
LrpCBA pilus-mediated cellular variation of endogenous IL-8 production in Caco-2 intestinal cells. Caco-2 cells were exposed to normalized cultures of *L*. *ruminis* cells and the recombinant GRS1224 (WT) and GRS1225 (LrpC-deleted) lactococci (MOI 100), with spent cell culture supernatants then measured for any variation in the levels of endogenous IL-8 cytokine production. GRS71 and GRS1052 cells were included as controls and treated likewise. Measurements were done in triplicate and limit bars indicate the SEM. Statistical differences for individual pairwise comparisons against the GRS71 control are designated as * = *P* ≤ 0.05 (significant) or *ns* = *P* > 0.05 (not significant).

Additionally, we tried to assess whether LrpCBA piliation exerts a modulatory effect on the cytokine production profile of non-recombinant host-immune cells. For this, we examined the intestinal Caco-2 cell line and monitored any changes to its endogenic release of pro-inflammatory IL-8 (Fig 10). Here, it seemed that once Caco-2 cells were exposed to the LrpCBA-piliated GRS1224 and GRS1225 clones there is some moderate lowering of IL-8 secretion as compared to those levels stimulated by the control cells (GRS71 and GRS1052). We interpret this as a dampening influence, which itself is in line with the results for the induced IL-8 modulation in HEK-TLR2 cells (see above). As well, this waning effect also mirrors that which we obtained previously in our immuno-analysis of the *L*. *rhamnosus* GG SpaFED pilus [14]. However, we again see that the LrpC adhesin pilin offers no causal contribution to the correspondingly diminished immune response (Fig 10). On this basis, it is reasonable to suggest that the innate immunogenic effects determined herein for the LrpCBA pilus are stemming more from the recognition of its overall macromolecular form and relying less on the functionality of its adhesive characteristics. Previously, we showed that the immuno-altering behavior to potentiate (via SpaCBA pili) or dampen (via SpaFED pili) had seemed to depend on the presence of ancillary tip pilins (i.e., SpaC and SpaF, respectively) [11,14], and then presumably where the adhesiveness of the subunits is key to the outcome. While the niche-fitness of SpaCBA and SpaFED pili to mutually adhere mucus might be reconciled as conducive to the underlying substrate specificity of the immune-related interactions, this would not be the case for the *L*. *ruminis* LrpCBA pilus, as it appears not to possess this particular binding functionality.

A notable conundrum with our obtained results lies in the minimal augmentation that *L*. *ruminis* cells have on the IL-8 secretion from Caco-2 cells (Fig 10). This is despite an earlier study having had attributed an increased release of IL-8 from intestinal epithelial cells, and where recognition between TLR5 and *L*. *ruminis* flagella is considered a key triggering interaction [24]. Irrespective of this discrepancy, in the broader scenario of bacterial recognition and host cell immune responsiveness within the gut, the *L*. *ruminis* species would seem to have adapted the concomitant ability to vary the extent of intestinal IL-8 secretion by host cells, either lowering it by way of surface pili (herein described) or raising it through specific flagellar components [24]. Speculatively, it is conceivable that such immunogenic see-sawing activity might suffice as a particularly localized mode of homeostatic counterbalancing that then operates to help maintain a natural and steady state of host immune tolerance toward *L*. *ruminis*, which itself would be favorable to the long-term intestinal survival of this commensalic bacterium.

Then again, our attempt to reveal a similar pattern of LrpCBA pilus-induced cytokine-dampening in so-called professional antigen-presenting cells was less compelling (S5 Fig). Here, we observed that after treating human moDCs with the LrpCBA-piliated GRS1224 lactococcal clone there is no significant lowering of the pro-inflammatory TNF-α and IL-12 or anti-inflammatory IL-10 responses. In fact, the measured level of production of each cytokine, on average and with error considered, is nearly equivalent following stimulation by the GRS1225, GRS1052, and GRS71 cells. In addition, although in our experiment *L*. *ruminis* cells would appear to elicit the most elevated release of the three DC-cytokines, the accompanying results provide no persuasive evidence to implicate LrpCBA piliation as being even partially responsible for any potentiating-like activity. In this regard, our previous study of the *L*. *rhamnosus* GG SpaFED pilus had yielded somewhat similar results [14]. Consequently, we have become more convinced that the blood-derived moDC-type does not adequately mimic certain effector-triggered immune responses from the gut mucosa epithelium, and then for at least our purposes of comparatively evaluating the molecular basis of pilus-mediated immunogenicity, it would seem a non-representative and less fitting cellular target. Moreover, an additional factor that contributes to the general unsuitability of moDCs is derived from their inherent but problematic donor-to-donor variation, as this tends to generate an overall skewing effect from any pooled data, which inevitably can lead to possibly distorted and biased cytokine response patterns.

## Conclusions

Although not at all yet fully understood, it is largely assumed that the biological premise lying behind the gut autochthony of many so-defined commensalic bacteria likely rests with a variety of different adaptive traits working in concert at the physiological, functional, and structural levels. Correspondingly, it is then through the ensuing interplay of accumulative and combined cellular effects that help foster what is perceived to be a continuous but peaceful coexistence with the host. Among the many genotypes and phenotypes seemingly involved are those enabling some intestine-inhabiting genera and species to thrive and do well ecologically in certain focalized niche locales. In this respect, *L*. *ruminis* can be considered an ideal paradigm for studying the molecular underpinnings that lead to such autochthonous colonization. For instance, as *L*. *ruminis* displays the phenotypes associated with motility and aero-intolerance, these presumably can then allow this gut-adapted commensal to propel its way into the more oxygen-limiting recesses of the layered mucosal epithelium. By comparison, the *modus operandi* of various invading intestinal pathogens is in itself generally quite similar [46]. Nonetheless, whilst in these surroundings, it could be expected that specific bacterial surface morphologies and features having functionalities for adherence will then promote *L*. *ruminis* attachment and retention, thereafter to be followed by cell growth and a sustained occupation of the gut.

In our present study of *L*. *ruminis*, we delved into unveiling certain aspects of a surface feature with a purported involvement in cellular adhesion functioning and immuno-signaling. Here, we demonstrated that the human intestinal *L*. *ruminis* strain ATCC 25644 displays constitutively expressed surface piliation, which itself derives genomically from the *lrpCBA*-called pilus-encoding operon. As similarly found with other piliated Gram-positive bacteria, these so-called LrpCBA pili are characterized as a sortase-dependent assemblage of three different protein subunits, namely the LrpC, LrpB, and LrpA pilins. Moreover, the larger sized ancillary LrpC subunit heeds functional precedent by helping confer a pilus-sourced adhesiveness that then supports *L*. *ruminis* binding to ECM-related substrates and gut epithelial cells, but otherwise not to mucosal surfaces. As also inferred from our results, it would seem somewhat apparent that once becoming host cell-recognized the LrpCBA pilus could evoke an immune-dampening effect in the form of diminished TLR2 signaling and IL-8 responsiveness, for which there conceivably might be a homeostatic role in modulating basal immune tolerance toward *L*. *ruminis* while it takes up residency in the intestine. Thus, when seen in the context of what helps establish *L*. *ruminis* indigeneity, LrpCBA piliation undoubtedly embodies an advantageous cell-surface phenotype, and so represents a functional accompaniment to that being provided by flagella. Yet clearly, surface piliation is just one facet of the many intertwined molecular intricacies (but as well metabolic processes) that are operating to uphold the stable and long-term gut colonization by *L*. *ruminis*. In this regard, our findings have only begun to scratch the “*in vitro* surface” in terms of what ecological associations lie between the *L*. *ruminis* species and the mammalian host intestine. However, in the future, to address whether LrpCBA pilus-mediated interactions exert any natural cellular outcome or impact regarding autochthonous behavior, this will necessitate pursuing microbe-host colonization studies that entail a more *in vivo*-based experimental approach.

## Materials and Methods

### Cultivation of bacterial strains and clones

The *L*. *lactis* NZ9000 (*pepN*::*nisRnisK*) strain [47] (relabeled in this study as GRS71) was employed as the cloning and expression host for recombinant *L*. *ruminis* LrpCBA pilus production. Cell growth was ordinarily carried out overnight at 30°C in M17 medium (Difco) and 0.5% glucose (GM17) using either an agar plate or a standing liquid broth. GM17 liquid medium was supplemented with 7.5 μg/ml chloramphenicol when cultivating the recombinant GRS1052, GRS1185 [11], GRS1189 [14], GRS1211 [11], GRS1224, GRS1225, and GRS1226 [14] lactococcal clones. Anaerobic growth of *L*. *ruminis* (ATCC 25644) cells was normally done overnight at 37°C with solid or liquid de Man-Rogosa-Sharpe (MRS) medium (Difco). *L*. *rhamnosus* GG (ATCC 53103) served as a control strain and was grown likewise in MRS medium.

### Culturing of cell lines

HEK-Blue^™^-hTLR2 (relabeled in this study as HEK-TLR2) is a commercially available recombinant cell line (purchased from InvivoGen), wherein HEK293 cells have been modified to contain the loci for human TLR2 and a NF-κB-inducible SEAP (secreted embryonic alkaline phosphatase) reporter system, with the latter used for TLR2 expression monitoring. HEK-TLR2 cells were grown in Dulbecco's modified Eagle’s medium (DMEM) containing 4.5 g/l glucose, along with 10% (v/v) heat-inactivated fetal calf serum (FCS), 2 mM L-glutamine, 100 μg/ml Normocin^™^, 50 U/ml penicillin, and 50 μg/ml streptomycin. HEK-Blue^™^ selection antibiotics were added to this culture medium only when deemed necessary. Cell culturing conditions were as specified by the manufacturer or as described previously [11]. Caco-2 cells [48] were obtained from our in-house culture collection and maintained in Roswell Park Memorial Institute (RPMI) 1640 medium containing 25 mM HEPES buffer and 2.2 mg/ml NaHCO_3_, and supplemented with 10% FCS, 2 mM L-glutamine, 50 μg/ml gentamicin, and 1% nonessential amino acids. HT-29 cells were purchased from the Culture Collections of Public Health England (Department of Health, United Kingdom) and cultured in McCoy’s 5A modified medium containing 10% FCS, 2 mM L-glutamine, 50 U/ml penicillin, and 50 μg/ml streptomycin. The Caco-2 and HT-29 cell lines originate from human colorectal adenocarcinoma cells, each having typical epithelial morphologies, although fully differentiated HT-29 cells tend to exhibit higher mucus secretion. Typically, Caco-2 and HT-29 cells were incubated for 21 and 14 days (respectively) in a humidified 5% CO_2_ atmosphere at 37°C, and once reaching a confluence of 90% they were seeded out for experiments. All culture media and reagents were procured from Gibco or Sigma unless otherwise indicated.

### Recombinant DNA manipulations

Commercially available kits and protocols were used to isolate and recover DNA from *L*. *ruminis* (genomic) and *L*. *lactis* or *E*. *coli* (plasmid) cells as recommended by the manufacturer, albeit with a few minor procedural changes. Unless otherwise so specified, conventional DNA methodologies and approaches (e.g., PCR amplification, restriction endonuclease digestion, DNA ligation, etc) were used for molecular cloning in *L*. *lactis* and *E*. *coli*. The pET28b+ expression vector (Novagen) was used for *E*. *coli* cloning. Expression vector pKTH5080 (unpublished) was used for lactococcal cloning and stems originally from the pNZ8032 plasmid [49], which encodes the regulatory loci (*nisR* and *nisK*) and promoter region (P*nisA*) for nisin-inducible protein production. For our purposes, the pNZ8032 plasmid had been modified previously to include the DNA sequences for the secretion signal and transcriptional termination sites of *Lactobacillus brevis* S-layer protein gene (*slpA*) [50], along with two new restriction endonucleases (*Eco*RI and *Xho*I) in the multiple cloning region.

### Construction of plasmids that express *L*. *ruminis* LrpCBA piliation in *L*. *lactis*

Construction of *Lactococcus*-propagating plasmids encoding for either WT or LrpC-deleted LrpCBA pilus production was done by using overlap extension PCR (OE-PCR) [51] and conventional cloning methodologies. For this work, the genome from *L*. *ruminis* ATCC 25644 (http://www.ncbi.nlm.nih.gov/genome/genomes/1955; BioProjects: PRJNA55509 and PRJNA31499) served as the source of the *lrpCBA*-called pilus operon. To create the WT LrpCBA pilus-bearing plasmid, this required that several sequential PCR amplifications be undertaken to build up the concatenated DNA sequence spanning the four ORFs of the fimbrial *lrpCBA* operon, i.e., HMPREF0542_RS04840 (l*rpC*), HMPREF0542_RS04845 (*lrpB*), HMPREF0542_RS04850 (*lrpA*), and HMPREF0542_RS04855 (*srtC*). In the first two PCR amplifications, two sets of paired forward and reverse oligonucleotide primers (i.e., 5'-GAAATAGAATAAAGACAAGCACATTACTGTAATAGTG with 5'-GTCGAACTTCCCCATTGCAAAAACAATCG and 5'-CGATTGTTTTTGCAATGGGGAAGTTCGAC with 5'-GATCGAAGAATTTACCTTGTTTCCGGTCTG; Oligomer Oy, Finland) were used to generate flanking upstream and downstream (~0.5 and ~3.6 kb, respectively) OE-PCR fragments. Taken together, these fragments encompass the entire *lrpC* gene (including its own secretion signal) along with a one-third upstream portion the *lrpB* gene. To eliminate an unwanted *Nco*I restriction site within the *lrpC* coding region, two of the primers contained nucleotide changes to incorporate the necessary point mutations (sites underlined in primers shown above). In one more PCR amplification, a flanking downstream OE-PCR fragment (~3.9 kb) with sequence covering the other two-thirds of *lrpB*, and as well the entire length of the *lrpA* and *srtC* genes, was generated using two other paired primers (5'-CAGACCGGAAACAAGGTAAATTCTTCGATC and 5'-GTCCGTAATCGCCATTTTTGGAGCAG). In the final PCR step, the three OE-PCR fragments were then amplified and joined together in-frame to produce a ~7.44 kb fragment by using forward and reverse primers that then also tag the ends with 5' *Nco*I and 3' *Xho*I sites (5'-GAGCAACTGCCATGGAAAGAAATAAAATATTTAAAAAG and 5'-CATAGTGTTTTCTCGAGCTTTTTTCAGCTAATG, respectively; restriction sites are underlined). To construct the LrpC-deleted plasmid, only a single length of DNA sequence encoding *lrpB*, *lrpA*, and *srtC* loci (but not *lrpC*) was PCR amplified using a different *Nco*I-containing forward primer (5'-AGGGGGTGTGACCATGGAGAGAGTATTAAAATTATTG; *Nco*I site is underlined), but with the same *Xho*I-containing reverse primer just mentioned above. Subsequent to their digestion by the *Nco*I and *Xho*I restriction endonucleases, the WT and LrpC-deleted *lrpCBA* operon-encoding PCR fragments were each ligated into a nisin-inducible expression plasmid (pKTH5080) and then transformed into electrocompetent *L*. *lactis* NZ9000 (GRS71) cells according to the procedure described previously [52]. Cells growing on solid GM17 growth medium supplemented with 7.5 μg/ml chloramphenicol were identified as transformant clones, with PCR analysis done to confirm whether they are carrying plasmids with the right-sized inserts. The so-named GRS1224 and GRS1225 lactococcal clones contain the plasmids that express WT (pKTH5441) and LrpC-deleted (pKTH5442) *L*. *ruminis* LrpCBA pili, respectively. The previously produced *L*. *lactis* GRS1052 clone (unpublished) carries the non-inserted pKTH5080 plasmid and was utilized as a control in this study.

### Nisin-induced expression in recombinant *L*. *lactis*

Overnight cultures of recombinant lactococcal clones (GRS1052, GRS1185, GRS1189, GRS1211, GRS1224, GRS1225, and GRS1226) were incubated statically at 30°C in liquid GM17 medium supplemented with chloramphenicol (7.5 μg/ml), and after being diluted 1:25 with fresh medium cell growth was continued until reaching an optical density at 600 nm (OD600) of ~0.5, at which point nisin was added to induce recombinant LrpCBA pilus production. Cells were incubated overnight at 30°C, centrifugally harvested and washed with phosphate-buffered saline (PBS) (pH 7.2), and then resuspended in cell culture medium, PBS, or SDS-PAGE loading buffer. The empty-vector GRS1052 clone was used as a control and treated similarly. The nisin-overexpressing *L*. *lactis* NZ9700 strain [53] (kindly supplied by Dr. François Douillard at the University of Helsinki) served as the nisin source and was added in the form of a 0.2% aliquot of a sterile-filtered cell-free supernatant that had been prepared from an overnight grown culture.

### Generation of antiserum against recombinant *L*. *ruminis* LrpCBA pilin-proteins

To generate antiserum against the *L*. *ruminis* LrpC, LrpB, and LrpA pilin subunits, purified recombinant versions of each protein were used. Here, cloning and expression of LrpCBA pilin-proteins in *E*. *coli* strains (TOP10 and BL21(DE3)/pLysS, respectively) was similarly carried out as described in our previous work [6]. Briefly, the coding sequence for the *lrpC*, *lrpB*, and *lrpA* loci was derived from the *L*. *ruminis* ATCC 25644 genome, with each being PCR amplified as a single-length fragment using pairs of flanking primers (Oligomer Oy, Finland) that then add 5' *Sac*I and 3' *Xho*I restriction sites at the ends. For this, the corresponding forward and reverse primers are 5'-CGAAAAGGCAAAGAGCTCGGTTGAGTTG and 5'-CATGCCGCCCTCGAGTGGAAGGAAG for *lrpC*, 5'-TCCGGGCAAGCATCGAGCTCTTCGACAAC and 5'-GCTTCACCCTCGAGCGGCAACATCC for *lrpB*, and 5'-TCAAGACCGCACAGAGCTCAGAAACGTCAAC and 5'-CATGCCGCCCTCGAGTGGAAGGAAG for *lrpA* (restriction sites are underlined). Following digestion by the *Sac*I and *Xho*I restriction endonucleases, each of the PCR products was ligated into the pET28b+ vector, whereupon when propagated and isopropyl β-D-1-thiogalactopyranoside-induced in *E*. *coli* cells the resultant plasmids (pKTH5428 for LrpC, pKTH5427 for LrpB, and pKTH5425 for LrpA) then produce intracellular pilin-proteins that lack N-terminal secretion signaling and C-terminal sortase recognition domains. Instead, these recombinant LrpCBA pilins are engineered to have a hexahistidyl tail at the COOH-terminus, but which also include some extra residues at the N-terminus (MGRDPNSSS) and ahead of the hexahistidines (LE), these all stemming from the *E*. *coli* cloning vector. Isolation of the recombinant LrpCBA pilins was by Ni^2+^-chelating affinity purification using the procedures and conditions essentially as outlined in our earlier studies [6,54]. LrpC (~123 kDa), LrpB (~39 kDa), and LrpA (~49 kDa) pilin-proteins were equilibrated in 10 mM Tris-HCl (pH 8.0) buffer, with purity reasonably close to homogeneity as judged by SDS-PAGE. Absorbance measurements at 280 nm were taken to estimate their protein concentrations. Eurogentec (Seraing, Belgium) produced polyclonal antiserum against recombinant LrpCBA pilin-proteins in rabbits as a fee-for-service using a standard 28-day immunization protocol (for additional details, see: www.eurogentec.com). For use in EM experiments, anti-LrpA serum also underwent IgG fractionation via an established method involving ammonium sulfate precipitation [55].

### Immunoblot detection analysis

Cells were grown overnight with solid (*L*. *ruminis*) or liquid (*L*. *rhamnosus* GG and nisin-induced recombinant lactococci) medium, harvested by scraping or centrifugation, rinsed at least once with PBS, and then resuspended with 10 mM Tris-HCl (pH 6.8) buffer. Cell suspensions were mixed 3:5 with 5 × gel loading buffer (135 mM Tris-HCl, pH 6.8, 30% glycerol, 3% SDS, and 0.03% bromophenol blue) and sonicated briefly, before being boiled (~5 minutes) and then centrifugally clarified. Protein content of the cell suspensions was resolved electrophoretically on precast Bio-Rad SDS-gradient gels (4–20%) and then transferred onto Immobilon^®^-P (polyvinylidene difluoride) membranes (Millipore). Afterward, to detect each of the *L*. *ruminis* LrpC, LrpB, and LrpA pilin-proteins, membranes were probed first with the different anti-LrpCBA pilin sera (diluted 1:2000 for anti-LrpB and 1:2500 for both anti-LrpA and anti-LrpC) and then with a 1:60,000 dilution of horseradish peroxidase-conjugated goat anti-rabbit IgG (Bio-Rad). Chemiluminescent visualization of pilus-related proteins was through the Amersham ECL^™^ Advance Western Blotting Detection Kit (GE Healthcare) as recommended in the manufacturer’s instructions.

### Immunogold labeling and electron microscopy

Immunogold pilin-protein labeling and electron microscopic analysis of bacterial cells were performed as had been described by Chang *et al* [56], along with some minor modifications. Cells cultivated overnight with solid (*L*. *ruminis*) or liquid (*L*. *rhamnosus* GG and nisin-induced recombinant lactococci) medium were harvested and then rinsed and resuspended with PBS. Single and double labeling EM experiments with formvar-carbon-coated copper grids involved the sequential use of rabbit antisera against *E*. *coli*-produced LrpCBA pilin-proteins (each diluted 1:2 to 1:8), protein A conjugated to gold particles of 10- and 15-nm size (each diluted 1:20), and negative staining (methylcellulose-uranyl acetate) solution. Highly magnified imaging of the grids was with a JEOL JEM-1400 transmission electron microscope on hand at the Institute of Biotechnology Electron Microscopy Unit (University of Helsinki).

### *In vitro* cellular adhesion to collagen, fibronectin, and intestinal epithelial cells

Measuring the ability of *L*. *ruminis* and lactococcal cells to adhere collagen and fibronectin proteins, and as well two intestinal epithelial cell lines (Caco-2 and HT-29), was done using a microplate-based assay method as described previously [14]. To quantify *in vitro* binding to types I and IV collagen and fibronectin (Sigma), 1-μg amounts of each protein were first thawed overnight while refrigerated and then coated into the wells of 96-well microplates (Corning) and allowed to incubate overnight at 4°C. Any remaining sites in these protein-coated wells were subsequently blocked by adding a solution of 5% skimmed milk and incubating for 2 hours at room temperature. After the corresponding bacterial cells had been metabolically radiolabeled using tritiated thymidine, and once their cell numbers had been normalized to an OD600 of ~0.5 with PBS buffer, they were added (100 μl per well) to the collagen- and fibronectin-precoated plates and incubated at room temperature for 2 hours. Each of the wells was next rinsed carefully with PBS at least three times to remove any unattached or lightly bound bacterial cells, with a 600-μl volume of lysis solution (1% SDS-0.1 N NaOH) added afterward and an overnight incubation at 37°C then continued. The cell suspension lysates were then recovered and mixed with 1-ml volumes of OptiPhase “HiSafe” III scintillation fluid (Perkin-Elmer Life Sciences), whereupon radioactive counting was performed with a liquid-scintillator detector. To assay adherence to the Caco-2 and HT-29 cell lines, confluent cells (prepared as described above) were first reseeded at a density of 1.0 × 10^4^ cells/well in 24-well culture plates (Corning) and then allowed to grow 21 (for Caco-2) or 14 (for HT-29) days at 37°C with 5% CO_2_ content. After each well had been washed two times with FCS-free culture medium, a 600-μl aliquot of radiolabeled bacterial cells (adjusted beforehand to OD600 of ~0.5 in the same cell culture medium) was then added and followed by a 2-hour incubation using the same growing conditions just mentioned above. The next steps of this assay are the same as those used for measuring binding to collagen and fibronectin. In both assays, cellular adhesion is calculated as a percent of the radioactivity in the wells and equates to the measured amounts in the cell suspension lysates relative to that associated with the cell suspensions at the outset.

### Biofilm formation assay

Static biofilm formation was assayed by crystal violet staining as earlier described [57], albeit with minor modifications. Overnight cultures of *L*. *lactis* NZ9000 (GRS71), nisin-induced recombinant lactococci (GRS1052, GRS1185, GRS1189, GRS1211, GRS1224, GRS1225, and GRS1226), *L*. *ruminis*, and *L*. *rhamnosus* GG were grown and prepared just as described in previous sections (see above). Each of these cultures was then normalized according to cell numbers and after that diluted 1:50 in a fresh aliquot of their respective growth medium. A 200-μl volume of each diluted culture was added to the wells of a 96-well microplate, which was then incubated without shaking for 24 hours, either aerobically at 30°C for the lactococcal cells or anaerobically at 37°C for the *L*. *ruminis* and *L*. *rhamnosus* GG cells. Afterward, the spent growth medium was aspirated away and the retained bacterial cells were rinsed twice with PBS (200 μl/well) before a 100-μl volume of 0.1% (w/v) crystal violet solution (made in MilliQ water) was added and then allowed to stain for 15 minutes at room temperature. The staining solution was then removed from each well, with any residual traces of stain being rinsed away with three 200-μl PBS washes. To solubilize the crystal violet from the stained bacterial cells, a 33% acetic acid solution (200 μl/well) was added and then allowed to incubate for 15 minutes at room temperature. Finally, to then determine the level of biofilm formation, the absorbance at 590 nm of the crystal violet recovered from each well was measured spectrophotometrically using a PerkinElmer VICTOR^3^ plate reader.

### Induction of immune signaling in HEK-TLR2 cells

Measurement of TLR2-mediated NF-kB activation in human HEK-TLR2 cells as induced by *L*. *ruminis* and LrpCBA-piliated lactococci was carried out as in our prior studies [11,14]. Using 24-well culture plates, HEK-TLR2 cells were seeded at about 5.0 × 10^4^ cells/well and subjected to an overnight incubation using the culture medium (devoid of selection antibiotics) and conditions already mentioned previously (see earlier section above). HEK-TLR2 cells were then overlaid with bacterial cells at a multiplicity of infection (MOI) of 100 and incubated overnight at 37°C. On the following day, each of the cell culture supernatants was analyzed for NF-kB activation as induced SEAP production, in which a 20-μl aliquot was taken and added to 180 μl of pre-warmed QUANTI-Blue reagent in a 96-well microplate, and after that incubated at 37°C. Levels of SEAP activity were determined using QUANTI-Blue^™^ detection medium in accordance with the manufacturer’s instructions, with the color development monitored spectrophotometrically at 620 nm. Measurements were made at different time intervals and in triplicate. Induced HEK-TLR2 cells were assayed for IL-8 production using the BD OptEIA^™^ ELISA kit by following the recommended instructions of the manufacturer (BD Biosciences). Other experiments performed that either involved heat treatments (100°C for 10 minutes) or using Transwell cell culture membrane inserts (Becton Dickinson & Company; 0.4-μm pore size) were done as described previously [11,14].

### Induced alterations to endogenous IL-8 production in Caco-2 cells

The Caco-2 cell line was cultured and readied using the conditions described beforehand (see earlier section above). Any variations to the endogenous IL-8 levels in Caco-2 cells that are caused by the added presence of *L*. *ruminis* and LrpCBA-piliated lactococci were assessed by analyzing each of the corresponding cell culture supernatants with the BD OptEIA^™^ ELISA kit using the instructions supplied by the manufacturer (BD Biosciences).

### Statistical data analysis

Certain data from this study was assessed statistically through the use of the GraphPad Prism statistical software package (version 4.0). *P*-values for pairwise comparisons were calculated with the unpaired Student’s *t* test and denoted as not significant (more than 0.05), significant (0.05 or less), very significant (0.01 or less), or highly significant (0.001 or less).

## Supporting Information

S1 Fig Primary structure comparison of the *L*. *ruminis lrpCBA* operon-encoded proteins. Amino acid sequences of the LrpC **(A)**, LrpB, **(B)**, LrpA **(C)**, and SrtC **(D)** proteins were deduced from the genomes of the *L*. *ruminis* ATCC 25644, SPM0211, ATCC 27782, DPC 6832, and GRL1172 strains. Multiple alignments for each of the protein types were assembled with the MultAlin program [58] (http://multalin.toulouse.inra.fr/multalin/multalin.html). Residues that match the consensus sequence (i.e., those amino acids in 100% of the primary structures) are denoted in red, whereas those residues not in the consensus sequence are denoted in black. Conservative amino acid replacements in the consensus sequence are identified by symbols (!, #, or $). Shown as an inset are percent identity matrices for each *L*. *ruminis lrpCBA* operon-encoded protein, which were calculated by Clustal2.1 (with default settings) using Clustal Omega at http://www.ebi.ac.uk/Tools/msa/clustalo/. (It should be noted that because a reading-frameshift change exists for the deduced primary structure of the DPC 6832-derived LrpB pili-protein, “X” has been added to its aligned sequence in place of a non-encoded serine residue. Based upon our analysis, while this seems to stem from an absent cytosine in the corresponding codon, it remains uncertain whether this is due to an authentic indel mutation, or simply caused by inaccurate DNA sequencing.)
(PDF)

S2 Fig Predictive detection of functional domains in the primary structure of *L*. *ruminis* (ATCC 25644) LrpCBA pilins by Pfam database searching. Primary structures of the LrpC **(A)**, LrpB **(B)**, and LrpA **(C)** pilin-proteins deduced from the *L*. *ruminis* ATCC 25644 genome were analyzed for hidden Markov model (HMM) matches by searching the Pfam database [28] at http://pfam.xfam.org. Predictive outputs of searches giving a significant Pfam-A match for each of the LrpCBA pilin-proteins are provided.
(PDF)

S3 Fig Multiple sequence alignment of the upstream regulatory region for the *L*. *ruminis lrpCBA* pilus operon. Shown is a multiple sequence alignment of 91 nucleotides (nt) that lie directly upstream from the first five codons of the *lrpC* gene (the first gene in the *L*. *ruminis* fimbrial *lrpCBA* operon). DNA sequence (106 nt) covering this region was taken from the genomes of the *L*. *ruminis* ATCC 25644, SPM0211, ATCC 27782, DPC 6832, and GRL1172 strains and aligned using MultAlin [58] (http://multalin.toulouse.inra.fr/multalin/multalin.html). Those nucleotides that are common to all sequences in the alignment, and thus that make up the consensus sequence, are denoted in red. Other nucleotides that are not part of the consensus sequence are denoted in black.
(PDF)

S4 Fig Immuno-electron microscopy of recombinant LrpCBA-piliated lactococci using preimmune serum controls. Immunogold labeling and electron microscopy of the WT GRS1224 (panels to the left) and LrpC pilin-deleted GRS1225 (panels to the right) lactococcal clones were carried out with preimmune serum as described in Materials and Methods. Preimmune serum of the same rabbit from which each pilin-specific antiserum had been raised was used for these experiments. Nisin-induced recombinant lactococci were single-labeled using the preimmune sera, Pre-LrpA (undiluted; top panels), Pre-LrpB (diluted 1:4; middle panels), and Pre-LrpC (diluted 1:8; bottom panels), along with protein A-10-nm gold particles. Representative EM images are shown. Scale bars are included in each panel.
(TIF)

S5 Fig LrpCBA pilus-mediated cellular stimulation of moDC-cytokine production. Human monocyte-derived dendritic cells (moDCs) were exposed to *L*. *ruminis* cells or the recombinant WT (GRS1224) and LrpC-deleted (GRS1225) lactococci and the subsequent stimulated production of the TNF-α **(A)**, IL-12 **(B)**, and IL-10 **(C)** cytokines was then measured using the same protocol from our previous published studies [11,14]. For this, overnight grown cultures were prepared (for details, see Materials and Methods) and then normalized according to OD600 in RPMI 1640 medium that contained 10% FCS, antibiotics, L-glutamine, and HEPES, but had no interleukin (IL)-4 and granulocyte-macrophage colony-stimulating factor (GM-CSF). Bacteria and moDCs were combined with a MOI of 50 and then incubated for about 24 hours at 37°C with 5% CO_2_ content. Cell culture supernatants were recovered and an estimation of the cytokine levels made by ELISA measurements using the BD OptEIA^™^ ELISA kit (BD Biosciences). The moDCs used in the experiments were generated from the blood of two different donors and then readied using essentially the same method as before [11,14]. GRS71 and GRS1052 cells, as well as RPMI cell-culture medium, were used as controls and treated as mentioned above. Triplicate measurements were made for each experiment, with limit bars showing the SEM. Statistical differences for individual pairwise comparisons against the GRS71 control are specified as ** = *P* ≤ 0.01 (very significant), * = *P* ≤ 0.05 (significant), or *ns* = *P* > 0.05 (not significant).
(TIF)
