# Supplementary material for: Human Gut-Commensalic Lactobacillus ruminis ATCC 25644 Displays Sortase-Assembled Surface Piliation: Phenotypic Characterization of Its Fimbrial Operon through In Silico Predictive Analysis and Recombinant Expression in Lactococcus lactis
Source: PLoS One. 2015 Dec 28;10(12):e0145718. doi: 10.1371/journal.pone.0145718 (PMC4692528; doi:10.1371/journal.pone.0145718)
Supplement: S1 Fig — Amino acid sequences of the LrpC (A), LrpB, (B), LrpA (C), and SrtC (D) proteins were deduced from the genomes of the L. ruminis ATCC 25644, SPM0211, ATCC 27782, DPC 6832, and GRL1172 strains. Multiple alignments for each of the protein types were assembled with the MultAlin program [58] (http://multalin.toulouse.inra.fr/multalin/multalin.html). Residues that match the consensus sequence (i.e., those amino acids in 100% of the primary structures) are denoted in red, whereas those residues not in the consensus sequence are denoted in black. Conservative amino acid replacements in the consensus sequence are identified by symbols (!, #, or $). Shown as an inset are percent identity matrices for each L. ruminis lrpCBA operon-encoded protein, which were calculated by Clustal2.1 (with default settings) using Clustal Omega at http://www.ebi.ac.uk/Tools/msa/clustalo/. (It should be noted that because a reading-frameshift change exists for the deduced primary structure of the DPC 6832-derived LrpB pili-protein, “X” has been added to its aligned sequence in place of a non-encoded serine residue. Based upon our analysis, while this seems to stem from an absent cytosine in the corresponding codon, it remains uncertain whether this is due to an authentic indel mutation, or simply caused by inaccurate DNA sequencing.) (PDF) [file pone.0145718.s001.pdf]

A

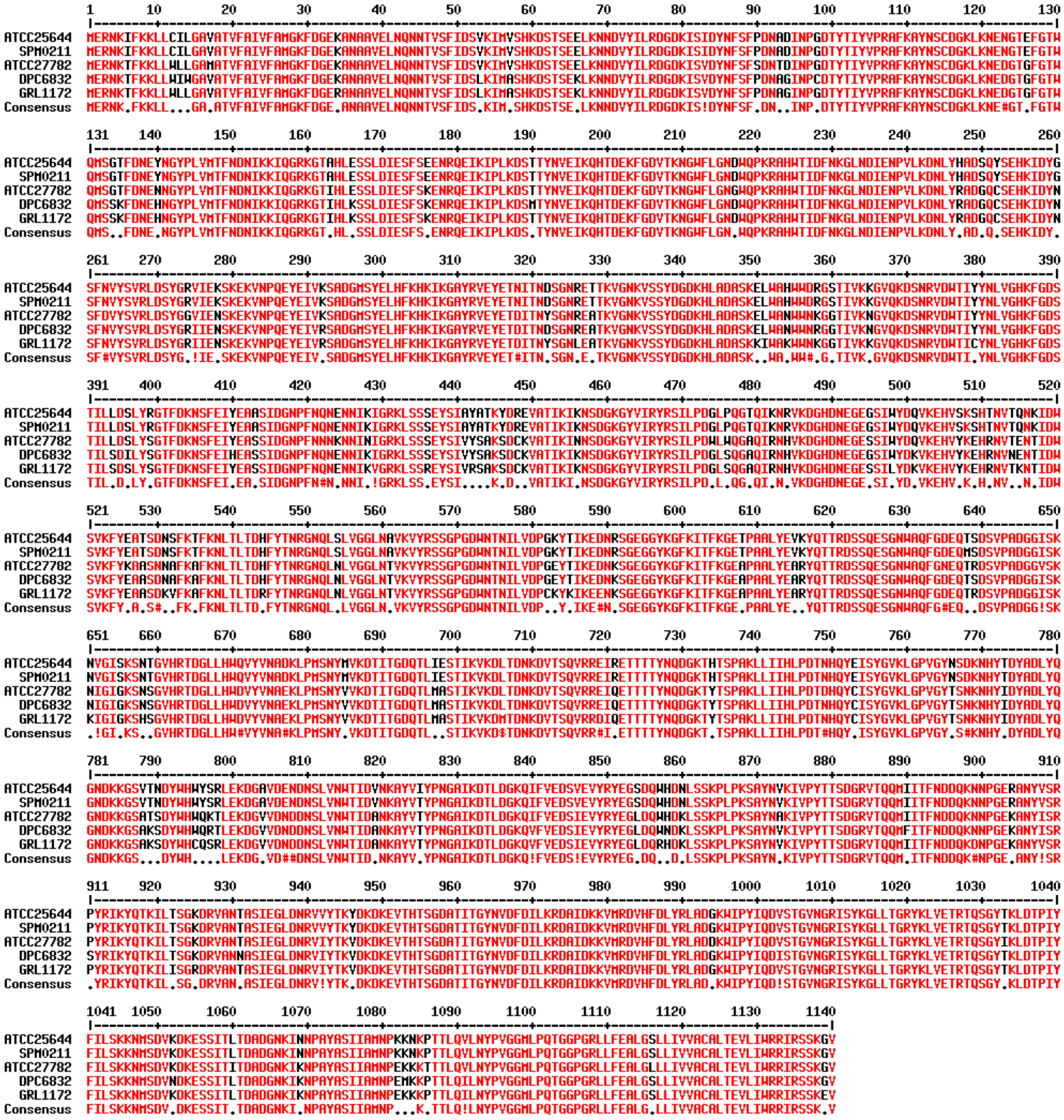

Percent Identity Matrix - created by Clustal2.1 (LrpC)

Strain	1	2	3	4	5
1: ATCC25644	100.00	99.82	90.61	89.65	90.61
2: SPM0211	99.82	100.00	90.61	89.65	90.61
3: ATCC27782	90.61	90.61	100.00	93.86	95.00
4: GRL1172	89.65	89.65	93.86	100.00	95.61
5: DPC6832	90.61	90.61	95.00	95.61	100.00

B

```
           1       10        20        30        40        50        60        70        80        90        100       110       120       130
           |--------+---------+---------+---------+---------+---------+---------+---------+---------+---------+---------+---------+---------|
ATCC25644  MKRVLKLLFMIVAFMTAVFAGSGQASADSTTGITQDIHIHISLGLNLPSGTDVTKLRAPTFEIYDISDQFNEADDPKEFTAKFPLGGQSYAKNFIEKHSLKPLSRQTGNKVNSSIDFIVPGCDAYLIVQT
  SPM0211           MIVAFMTAVFAGSGQASADSTTGITQDIHIHISLGLNLPSGTDVTKLRAPTFEIYDISDQFNEADDPKEFTAKFPLGGQSYAKNFIEKHSLKPLSRQTGNKVNSSIDFIVPGCDAYLIVQT
  GRL1172  MKKVLKLLFMIIAFMTAVFAGSGQASADSTTGITQNIHIHISLGLDLPSGTDVTKLRAPTFEIYDISDQFNEADDPKEFTEKFPLAGQSYAKNFIEKHSLKPLSRQTGNKVNSSIDFIVPGCDAYLIVQT
  DPC6832  MKKVLKLLFMIVAFMTAVFAGSGQASADSTTGITQNIHIHISLGLDLPSGTDVTKLRAPTFEIYDISDQFNEADDPKDFTEKFPLAGQSYAKNFIEKHSLKPLSRQTGNKANSSIDFIVPGCDAYLIVQT
ATCC27782                                            MGLNLPSGTDVTKLRAPTFEIYDISDQFNEADDPKEFTAKFPLVGQSYAKNFIEKHSLKPLSRQTGNKANSSIDFIVPRCDAYLIVQT
Consensus  ..........................................$GL#LPSGTDVTKLRAPTFEIYDISDQFNEADDPK#FT.KFPL.GQSYAKNFIEKHSLKPLSRQTGNK.NSSIDFIVP.CDAYLIVQT

           131     140       150       160       170       180       190       200       210       220       230       240       250       260
           |--------+---------+---------+---------+---------+---------+---------+---------+---------+---------+---------+---------+---------|
ATCC25644  DENGVIENAGNNGTFTLPFVFLMNDDFKIDDQGLMWFQIKGKTSLVQRSAYFFKYGKNAGGELPLSDAKFVLYRLDGSIKLYCTNNGGFKASASPLSDDEIAKFTSNSAGLVMYDRESLDSGTYYFSEVQ
  SPM0211  DENGVIENAGNNGTFTLPFVFLMNDDFKIDDQGLMWFQIKGKTSLVQRSAYFFKYGKNAGGELPLSDAKFVLYRLDGSIKLYCTNNGGFKASASPLSDDEIAKFTSNSAGLVMYDRESLDPGTYYFSEVQ
  GRL1172  DENGVIENAGNNGTFTLPFVFLMNDDFKIDDQGRMWFQIKGKTSLVQRSAYFFKYGKNAGGELPLSDAKFVLYRLDGSIKLYCTNNGGFKASASPLSDDEIAKFTSNSAGLVMYDRESLDPGTYYFSEVQ
  DPC6832  DENGVIENAGNNGTFTLPFVFLMNDDFKIDDQGRMWFQIKGKTSLVQRSAYFFKYGKNAGGELPLSDAKFVLYRLDGXIKLYCTNNGGFKACASPLSDDEIAKFTSNSAGLVMYDHESLDPGTYYFSEVQ
ATCC27782  DENGVIENAGDNGTFTLPFVFLMNDDFKIDDQGRMWFQIKGKTSLVQRSAYFFKYGKNAGGELPLSDAKFVLYRLDGSIKLYCTNNGGFKASASPLSDDEIAKFTSNSAGLVMYDRESLDPGTYYFSEVQ
Consensus  DENGVIENAG#NGTFTLPFVFLMNDDFKIDDQG.MWFQIKGKTSLVQRSAYFFKYGKNAGGELPLSDAKFVLYRLDG.IKLYCTNNGGFKA.ASPLSDDEIAKFTSNSAGLVMYD.ESLD.GTYYFSEVQ

           261     270       280       290       300       310       320       330       340       350       360       370       380       390
           |--------+---------+---------+---------+---------+---------+---------+---------+---------+---------+---------+---------+---------|
ATCC25644  APKGYRITDEARKIEVYIPKKLSDGVKVNGTALEELYDQKLSDGAVSAAKPRIYNYSIENPPSNSGKTNTPGKPNTPGKPNTSGKP------------------TTPNSVRKKGLWGMLPQTGEAKSIMA
  SPM0211  APKGYRITDEARKIEVYIPKKLSDGVKVNGTALEELYDQKLSDGAVSAAKPRIYNYSIENPPSNSGKTNTPGKPNTPGKPNTSDKP------------------TTPNSVRKKGLWGMLPQTGEAKSIMA
  GRL1172  APKGYRITDEARKIEVYIPKKMSDSVKVNGTALEELYDQKLSDGAVSAAKPRIYNYSIENPPGNSGKTNTPGNPNTPGKTNTPGNPNTPGKTNTPGNPNTPGNTTTPKSVKKKGLWGMLPQTGEAKSIMA
  DPC6832  APKGYRITDEARKIEVYIPKKMSDSVKVNGTALEELYDQKLSDGAVSAAKPRIYNYSIENPPSNSGKTNTPGNP------------------------------TTPKSVKKKGLWGMLPQTGEAKSIMA
ATCC27782  APKGYRITDEARKIEVYIPKKMSDGVKVNGTALEGLYDQKLSDGAVSAAKPRIYNYSIENPPSNSGKTNTPGKP------------------------------TTPKSVKKKGLWGMLPQTGESKSIMA
Consensus  APKGYRITDEARKIEVYIPKK$SD.VKVNGTALE.LYDQKLSDGAVSAAKPRIYNYSIENPP.NSGKTNTPG.P..............................TTP.SV.KKGLWGMLPQTGE.KSIMA

           391     400       410 414
           |--------+---------+---|
ATCC25644  LLGIGIICLVVLVSVGRRNYKEEH
  SPM0211  LLGIGIICLVVLVSVGRRNYKEEH
  GRL1172  LLGIGIICLVVLVSVERRNYNKEH
  DPC6832  LLGIGIICLVVLVSVERRNYNKEH
ATCC27782  LLGIGIICLVVLVSAKRRNYKEEH
Consensus  LLGIGIICLVVLVS..RRNY..EH
```

Percent Identity Matrix - created by Clustal2.1 (LrpB)

Strain	1	2	3	4	5
1: GRL1172	100.00	98.18	95.03	94.70	94.83
2: DPC6832	98.18	100.00	94.72	94.79	95.20
3: ATCC27782	95.03	94.72	100.00	95.91	96.20
4: ATCC25644	94.70	94.79	95.91	100.00	99.48
5: SPM0211	94.83	95.20	96.20	99.48	100.00

C

```
           1       10        20        30        40        50        60        70        80        90        100       110       120       130
           |--------+---------+---------+---------+---------+---------+---------+---------+---------+---------+---------+---------+---------|
ATCC25644  MKNHKKLRNALATLLLALPLALQGAVGVKTAQAAETSTETATVTLHKYVFDKSLPSDKIDNSKSQDEINAWLTKNNAEALDGVEFTAYDVTSEYADAYKTATGDKNESPADAAKTASAAVAKKADALQKT
  SPM0211  MKNHKKLRNALATLLLALPLALQGAVGVKTAQAAETSTETATVTLHKYVFDKSLPSDKIDNSKSQDEINAWLTKNNAEALDGVEFTAYDVTSEYADAYKTATGDKNESPADAAKTASAAVAKKADALQKT
ATCC27782  MKNHRKLRNALAILLLALPLALQGAVGVKTAQAAETSTETATVTLHKYVFDKSLPSDKIDNSKSQDEINAWLTKNNAEALDGVEFTAYDVTSEYANEYKTATGDKNESPADAAKTASAAVAKKADALQKT
  GRL1172  MKNHKKLRNALATLLLALPLALQGAVGVKTAQAAETSTETVTVTLHDYVSNKSLPFDKIDNSKSQDEINAWLTENNAEALDGVEFTAYDVTREYADAYETATGDKNESPADAAKTASAAVAKKADELQKT
  DPC6832  MKNHRKLRNALATLLLALPLALQGAVGVKTAQAAETSTETVNVTLHDYVSNKSLPFDKIDNSKSQDEINAWLTENNAEALDGVEFTAYDVTREYANAYETATGDKNESPADAAKTASAAVAEKADVLQKT
Consensus  MKNH.KLRNALA.LLLALPLALQGAVGVKTAQAAETSTET..VTLH.YV.#KSLP.DKIDNSKSQDEINAWLT.NNAEALDGVEFTAYDVT.EYA#.Y.TATGDKNESPADAAKTASAAVA.KAD.LQKT

           131     140       150       160       170       180       190       200       210       220       230       240       250       260
           |--------+---------+---------+---------+---------+---------+---------+---------+---------+---------+---------+---------+---------|
ATCC25644  AT--VVGKQTTANGGLASFANLPLRDANGNYKAYLFAETDAPANITQKAEPFVLAMPIYGADGKTVQKSINIYPKNVKQSDKKTLNDNRSHHDFTAGEKINYSIETVVPWNIANKKVYTITDNPSKGLIM
  SPM0211  AT--VVGKQTTANGGLASFANLPLRDANGNYKAYLFAETDAPANITQKAEPFVLAMPIYGADGKTVQKSINIYPKNVKQSDKKTLNDNRSHHDFTAGEKINYSIETVVPWNIANKKVYTITDNPSKGLIM
ATCC27782  AT--VVGKQTTANGGLASFANLPLRDANGNYKAYLFAETDAPANITQKAEPFVLAMPIYGADGKTVQKSINIYPKNVKQSDKKTLNDNRSHHDFTAGEKINYTIETVVPWNIANKKVYTITDNPSKGLIM
  GRL1172  ATTTVVGKQTTANGGLASFANLPLRDENGNYKAYLFVETDSPANVIQKAEPFVLAMPVYGADGKTVQKSINIYPKNVKQLDKKTLGEK--HKDFTAGEKIKYSIETVVPWNIGNKEVYTITDNPSKGLIM
  DPC6832  ATTTVVGKQTTANGGLASFANLPLRDANGNYKAYLFVETDSPANVTQKSEPFVLAMPVYGADGKTVQKSINIYPKNVKQLDKHTLKED--HKDFTAGEKIKYSIETVVPWNIANKKVYTITYNPIKGFIM
Consensus  AT..VVGKQTTANGGLASFANLPLRD.NGNYKAYLF.ETD.PAN!.QK.EPFVLAMP!YGADGKTVQKSINIYPKNVKQ.DK.TL.#...H.DFTAGEKI.Y.IETVVPWNI.NK.VYTIT.NP.KG.IM

           261     270       280       290       300       310       320       330       340       350       360       370       380       390
           |--------+---------+---------+---------+---------+---------+---------+---------+---------+---------+---------+---------+---------|
ATCC25644  DADTIQIEGLASNKYTVKKNADNGFTITIPAANLAAFAGKTLKTTVKGHLSIEDLTLIDTGIPNKATAKVDNEAHHEVKSEEVFTGGKKFVKVDGSNQSKTLAGAQFQLVIVKNGQVVKYAHGNEKDGYT
  SPM0211  DADTIQIEGLASNKYTVKKNADNGFTITIPAANLAAFAGKTLKTTVKGHLSIEDLTLIDTGIPNKATAKVDNEAHHEVKSEEVFTGGKKFVKVDGSNQSKTLAGAQFQLVIVKNGQVVKYAHGNEKDGYT
ATCC27782  DADTIQIEGLASNKYTVKKNADNGFTITIPAADLAAFAGKTLKTTVKGHLSIEDLTLIDTGIPNKATAKVDNEAHHEVKSEEVFTGGKKFVKVDGSNQSKTLAGAQFQLVIVKNGQVVKYAHGNEKDGYT
  GRL1172  DTDTIQIEGLASNKYTVDKNADNGFTITIPAADLAAFAGKTLKTTVKGHLSIEDLTLIETGIPNKATAKVDNEAHLEVKSEEVLTGGKKFVKVDGSNQSKTLAGAQFQLVIVKNGQVVKYAHGNEKDGYT
  DPC6832  DTDTIQIEGLSSNKYTVDKNADNGFTITIPAADLAAFAGKTLKTTVKGHLSIEDLTLIDTDIINKATAKVDNEAHHEVKSEGVFTGGKKFVKVDGSNQSKTLAGAQFQLVIVKNGQVVKYAHGNEKDGYT
Consensus  D.DTIQIEGL.SNKYTV.KNADNGFTITIPAA#LAAFAGKTLKTTVKGHLSIEDLTLI#T.I.NKATAKVDNEAH.EVKSE.V.TGGKKFVKVDGSNQSKTLAGAQFQLVIVKNGQVVKYAHGNEKDGYT

           391     400       410       420       430       440       450       460       470       480       490       500      509
           |--------+---------+---------+---------+---------+---------+---------+---------+---------+---------+---------+--------|
ATCC25644  FDTNNTNVATKTTGENGQFEFAGLKYSESLEAGESYAVKEVKAPTGYDLLKDPVLFTVTKDSYKTVQAADGQKISNTKKGGFLPSTGGMGIVLFIAAGVVVMAGAAGTMIVRRNRRENI
  SPM0211  FDTNNTNVATKTTGENGQFEFAGLKYSESLEAGESYAVKEVKAPTGYDLLKDPVLFTVTKDSYKTVQAADGQKISNTKKGGFLPSTGGMGIVLFIAAGVVVMAGAAGTMIVRRNRRENI
ATCC27782  FDTNNTNVVTKTTGENGQFEFAGLKYSESLEAGESYAVKEVKAPTGYDLLKDPVLFTVAKDSYKTVQAADGQKISNTKKGGFLPSTGGMGIVLFIVAGVVVMAGAAGTMIARRNRRENI
  GRL1172  FDTNNTNVATKTTGENGQFEFAGLKNSTELEAGESYAVKEVKAPTGYNLLKDPVLFTVEEGSYKTVQATDGQKISNTKKGGFLPSTGGMGIVLFIVAGVVVMAGAAGTMIARRNRHENI
  DPC6832  FDTNNTNVSTKTTGENGQFEFVGLKNSTELEAGESYAVKEVKAPAGYNLLNDPVLFTVEKGSYKTVQAADGQKISNTKKGGFLPSTGGMGIVLFIVAGVVVMVGAAGAMIARRNRRENI
Consensus  FDTNNTNV.TKTTGENGQFEF.GLK.S..LEAGESYAVKEVKAP.GY#LL.DPVLFTV...SYKTVQA.DGQKISNTKKGGFLPSTGGMGIVLFI.AGVVVM.GAAG.MI.RRNR.ENI
```

Percent Identity Matrix - created by Clustal2.1 (LrpA)

Strain	1	2	3	4	5
**1: ATCC25644**	100.00	100.00	98.03	92.08	90.30
**2: SPM0211**	100.00	100.00	98.03	92.08	90.30
**3: ATCC27782**	98.03	98.03	100.00	91.49	90.69
**4: GRL1172**	92.08	92.08	91.49	100.00	93.69
**5: DPC6832**	90.30	90.30	90.69	93.69	100.00

D

```
           1       10         20         30         40         50         60         70         80         90         100        110        120        130
ATCC25644  MAEKKKKSKNDFWIQVLLMIAFLAGLLIMLYPFYVESINNFIDNQRIEEAQKLDAKRNAKELAKLRAENERAAKKAAKDPFRGTGNMNAKKLRRHLLGRVVIPKINVNVPLFNLTTADTLNYGAAVLQGS
  SPM0211  MAEKKKKSKNDFWIQVLLMIAFLAGLLIMLYPFYVESINNFIDNQRIEEAQKLDAKRNAKELAKLRAENERAAKKAAKDPFRGTGNMNAKKLRRHLLGRVVIPKINVNVPLFNLTTADTLNYGAAVLQGS
ATCC27782  MAEKKKKSKNGFWIQVLLMIVFLAGLLIMLYPFYVESINNFIDNQRIEEAQKLDAKRNAKELAKLRAENERAAKKAAKDPFRGTDNMNAEKLRKHLLGRVVIPKINVNVPLFNLTTADTLNYGAAVLQGS
  GRL1172  MAEKKKKSKNGFWIQVLLMIVFLAGLLIMLYPFYVESINNFIDNQRIEESQKLDAKRNAKELAKLRAENERAAKKAAKDPFRGTGSMNAKKLRRHLLGRVVIPKINVNVPLFNLTTSDTLNYGAAVLQGS
  DPC6832  MAEKKKKSKNGFWIQVLLMIVFLAGLLIMLYPFYVEAINNFIDNQRIEESQKLDAKRNAKELAKLRAENERAAKKAAKDPFRGTGSMNAKKLRRHLLGRVVIPKINVNVPLFNLTTADTLNYGAAVLQGS
Consensus  MAEKKKKSKN.FWIQVLLMI.FLAGLLIMLYPFYVE.INNFIDNQRIEE.QKLDAKRNAKELAKLRAENERAAKKAAKDPFRGT..MNA.KLR.HLLGRVVIPKINVNVPLFNLTT.DTLNYGAAVLQGS

           131     140        150        160        170        180        190        200        210        220        230        240        250        260
ATCC25644  SFPTGGKGKRTVIAAHRGLPERKLFTDLDKVKKGDLFVISVYGKNMAYKVYNIKVIKPNKVKSLLPVKDKDLATLMTCTPYMINSHRMLVTGYRVPYTKKIAREVEGASLMNNLIQAAVMLGCVMAIFSV
  SPM0211  SFPTGGKGKRTVIAAHRGLPERKLFTDLDKVKKGDLFVISVYGKNMAYKVYNIKVIKPNKVKSLLPVKDKDLATLMTCTPYMINSHRMLVTGYRVPYTKKIAREVEGASLMNNLIQAAVMLGCVMAIFSV
ATCC27782  SFPTGGKGKRTVIAAHRGLPERKLFTDLDKVKKGDLFVISVYGKNMAYKVYNIKVIKPNKVKSLLPVKDKDLATLMTCTPYMINSHRMLVTGYRVPYTKKIAREVEGASLMNNLIQAAVMLGCVMAIFSV
  GRL1172  SFPTGGKGKRTVIAAHRGLPERKLFTDLDKVKKGDLFVISVYGKNMAYKVYNIKVIKPNKVKSLLPVKDKDLATLMTCTPYMINSHRMLVTGYRVPYTKKIAREVEGASLMNNLIQAAVMLGCVMAIFSV
  DPC6832  SFPTGGKGKRTVIAAHRGLPERKLFTDLDKVKKGDLFVISVYGKNMAYKVYNIKVIKPNKVKALLPVKDKDLATLMTCTPYMINSHRMLVTGYRVPYTKKIAREVEGASLMNNLIQAAVMLGCVMAIFSV
Consensus  SFPTGGKGKRTVIAAHRGLPERKLFTDLDKVKKGDLFVISVYGKNMAYKVYNIKVIKPNKVK.LLPVKDKDLATLMTCTPYMINSHRMLVTGYRVPYTKKIAREVEGASLMNNLIQAAVMLGCVMAIFSV

           261     270        280        290        300        310        320        330        340        350        360        370      38082
ATCC25644  FYILYRIIHGGLLKKREINLDFIVADADGKPVVGEAFQLFARNGRRKLYRNQKEFIVQSDEQGRVRFTNLPGNVYCIKNDHLSVRAGIKKLRQENAALYPKKKQKSFIAQDNEKIWIVKNHH
  SPM0211  FYILYRIIHGGLLKKREINLDFIVADADGKPVVGEAFQLFARNGRRKLYRNQKEFIVQSDEQGRVRFTNLPGNVYCIKNDHLSVRAGIKKLRQENAALYPKKKQKSFIAQDNEKIWIVKNHH
ATCC27782  FYILYRIIHGGLLKKREINLDFIVVDADGKPVVGEAFRLFARNGRRKLYRNQKEFIVQSDERGRVRFTNLPGNVYCIKNDHLSVRAGIKKLRQENAALYPKKKQKSFIAQDNEKIWIVKNHH
  GRL1172  FYILYRIIHGGLLKKREINLDFIVVDADGKPVVGEAFQLFARNGRRKLYRNQKEFIVQSDEQGRVRFTNLPGNVYCIKNDHLSVRAGIKKLRQENAALYPNKKQKSFIAQDNEKNWIVKNHH
  DPC6832  FYILYRIIHGGLLKKREINIDFIVVDADGKPVVGEAFQLFARNGRRKLYRNQKEFIVQSDEQGRVRFTNLPGNVYCIKNDHLSVRAGIKKLRQENAALYPKKKQKSFIAQNNEKNWIVKNHH
Consensus  FYILYRIIHGGLLKKREIN.DFIV.DADGKPVVGEAF.LFARNGRRKLYRNQKEFIVQSDE.GRVRFTNLPGNVYCIKNDHLSVRAGIKKLRQENAALYP.KKQKSFIAQ#NEK.WIVKNHH
```

Percent Identity Matrix - created by Clustal2.1 (SrtC)

Strain	1	2	3	4	5
1: GRL1172	100.00	98.43	97.91	97.91	97.38
2: DPC6832	98.43	100.00	97.38	97.38	96.86
3: ATCC25644	97.91	97.38	100.00	100.00	97.91
4: SPM0211	97.91	97.38	100.00	100.00	97.91
5: ATCC27782	97.38	96.86	97.91	97.91	100.00
