# Supplementary material for: Human Gut-Commensalic Lactobacillus ruminis ATCC 25644 Displays Sortase-Assembled Surface Piliation: Phenotypic Characterization of Its Fimbrial Operon through In Silico Predictive Analysis and Recombinant Expression in Lactococcus lactis
Source: PLoS One. 2015 Dec 28;10(12):e0145718. doi: 10.1371/journal.pone.0145718 (PMC4692528; doi:10.1371/journal.pone.0145718)
Supplement: S2 Fig — Primary structures of the LrpC (A), LrpB (B), and LrpA (C) pilin-proteins deduced from the L. ruminis ATCC 25644 genome were analyzed for hidden Markov model (HMM) matches by searching the Pfam database [28] at http://pfam.xfam.org. Predictive outputs of searches giving a significant Pfam-A match for each of the LrpCBA pilin-proteins are provided. (PDF) [file pone.0145718.s002.pdf]

A

Sequence search results

Show the detailed description of this results page.

We found **3** Pfam-A matches to your search sequence (**all** significant). You did not choose to search for Pfam-B matches.

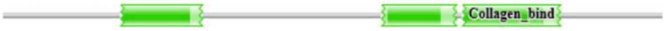

Hide the search options and sequence that you submitted.

You ran **one** job, a **Pfam-A** search with **ID** 12F236A8-E997-11E4-A1FF-E44495374F7B.

These are the options that you chose:

- You set the **Pfam-A** E-value cut-off to **1.0**

This is the **sequence** that you submitted:

```
MERNKIFKKLLCILGAVATVFAIVFAMGKFDGEKANAAVELNQNNTVSFIDSVKIMVSHKDSTSEELKNNDVY
ILRDGDKISIDYNFSFPDNADINPGDTYTIYVPRAFKAYNSCDGKLKNENGTEFGTWQMSGTFDNEYNGYPLV
MTFNDNIKKIQGRKGTAHLESSLDIESFSEENRQEIKIPLKDSTTYNVEIKQHTDEKFGDVTKNGWFLGNDWQ
PKRAHWTIDFNKGLNDIENPVLKDNLYHADSQYSEHKIDYGSFNVYSVRLDSYGRVIEKSKEKVNPQEYEIVK
SADGMSYELHFKHKIKGAYRVEYETNITNDSGNRETTKVGNKVSSYDGDKHLADASKELWAHWWDRGSTIVKK
GVQKDSNRVDWTIYYNLVGHKFGDSTILLDSLYRGTFDKNSFEIYEAASIDGNPFNQNENNIKIGRKLSSSEY
SIAYATKYDREVATIKIKNSDGKGYVIRYRSILPDGLPQGTQIKNRVKDGHDNEGEGSIWYDQVKEHVSKSHT
NVTQNKIDWSVKFYEATSDNSFKTFKNLTLTDHFYTNRGNQLSLVGGLNAVKVYRSSGPGDWNTNILVDPGKY
TIKEDNRSGEGGYKGFKITFKGETPAALYEVKYQTTRDSSQESGNWAQFGDEQTSDSVPADGGISKNVGISKS
NTGVHRTDGLLHWQVYVNADKLPMSNYMVKDTITGDQTLIESTIKVKDLTDNKDVTSQVRREIRETTTTYNQD
GKTHTSPAKLLIIHLPDTNHQYEISYGVKLGPVGYNSDKNHYTDYADLYQGNDKKGSVTNDYWHWYSRLEKDG
AVDENDNSLVNWTIDVNKAYVIYPNGAIKDTLDGKQIFVEDSVEVYRYEGSDQWHDNLSSKPLPKSAYNVKIV
PYTTSDGRVTQQMIITFNDDQKNNPGERANYVSRPYRIKYQTKILTSGKDRVANTASIEGLDNRVVYTKYDKD
KEVTHTSGDATITGYNVDFDILKRDAIDKKVMRDVHFDLYRLADGKWIPYIQDVSTGVNGRISYKGLLTGRYK
LVETRTQSGYTKLDTPIYFILSKKNMSDVKDKESSITLTDADGNKINNPAYASIIAMNPKKNKPTTLQVLNYP
VGGMLPQTGGPGRLLFEALGSLLIVVACALTEVLIWRRIRSSKGV
```

You can bookmark this URL to retrieve your results later:

http://pfam.xfam.org/search/sequence/results/12F236A8-E997-11E4-A1FF-E44495374F7B

Return to the search form to look for Pfam domains on a new sequence.

Significant Pfam-A Matches

Show or hide all alignments.

Family	Description	Entry type	Clan	Envelope Start	Envelope End	Alignment Start	Alignment End	HMM From	HMM To	HMM length	Bit score	E-value	Predicted active sites	Show/hide alignment
Collagen_bind	Collagen binding domain	Domain	CL0204	205	346	207	325	**4**	**114**	134	42.5	6.6e-11	n/a	Hide

```
#HMM       yyKsGdvdkedne..lvrWevniNnakeeienatltDt......igegqtltadsievaeenykkngevgsldevlseapgvksideatdngfkvtfggtidnavkisYktkitdsgkk
#MATCH     + K+G++  +d +  + +W++++N+ +++ien +l+D       + ++ +++++s++v+ + +++ g+v +  +++++ + + ++++a+     ++ f+  i+ a++++Y+t+it+ + +
#PP        78*****99998888**************98888765211111678899************************9999999**********************************87544
#SEQ       VTKNGWFLGNDWQpkRAHWTIDFNKGLNDIENPVLKDNlyhadsQYSEHKIDYGSFNVYSVRLDSYGRVIEKSKEKVNPQEYEIVKSADGMSYELHFKHKIKGAYRVEYETNITNDSGN
```

Family	Description	Entry type	Clan	Envelope Start	Envelope End	Alignment Start	Alignment End	HMM From	HMM To	HMM length	Bit score	E-value	Predicted active sites	Show/hide alignment
Collagen_bind	Collagen binding domain	Domain	CL0204	656	785	666	759	**16**	**107**	134	29.7	5.7e-07	n/a	Hide

```
#HMM       elvrWevniNnakeeienatltDtigegqtltadsievaeenykkngevgsldevlseapgvksideatdngfkvtfgg..tidnavkisYktk
#MATCH      l +W+v +N+ k ++ n  ++Dti+++qtl  ++i+v+ ++ +k+ + +   e    ++++++  ++ ++  k+ +      ++  + isY  k
#PP        479*************************************99997776655533333333333334444444444444778888888655
#SEQ       GLLHWQVYVNADKLPMSNYMVKDTITGDQTLIESTIKVKDLTDNKDVTSQVRREIRETTTTYNQDGKTHTSPAKLLIIHlpDTNHQYEISYGVK
```

Family	Description	Entry type	Clan	Envelope Start	Envelope End	Alignment Start	Alignment End	HMM From	HMM To	HMM length	Bit score	E-value	Predicted active sites	Show/hide alignment
Collagen_bind	Collagen binding domain	Domain	CL0204	796	965	798	936	**3**	**125**	134	37.5	2.3e-09	n/a	Hide

```
#HMM       vyyKsGdvdkednelvrWevniNnakeeienatltDtigegqtltadsievaeenykkngevgsldevls......eapgvksideatdngfkvtfgg...........tidnavkisYktkitdsgkkqsyknkatlng
#MATCH      ++K G vd++dn+lv+W++++N+a     n  ++Dt  ++q  + ds+ev++ + + +     + + l        +   + + d  ++   +tf++              + +++i+Y+tki +sgk   ++n+a ++g
#PP        579*************************************************97655553222222222254557777777888888899999**99999999999888899************999.9***996554
#SEQ       RLEKDGAVDENDNSLVNWTIDVNKAYVIYPNGAIKDTLDGKQIFVEDSVEVYRYEGSDQWHDNLSSKPLPksaynvKIVPYTTSDGRVTQQMIITFNDdqknnpgeranYVSRPYRIKYQTKILTSGKD-RVANTASIEG
```

B

Sequence search results

Show the detailed description of this results page.

We found **1** Pfam-A match to your search sequence (**all** significant). You did not choose to search for Pfam-B matches.

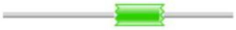

Hide the search options and sequence that you submitted.

You ran **one** job, a **Pfam-A** search with **ID** 8A550A80-E998-11E4-ADCF-D176298E2F76.

These are the options that you chose:

- You set the **Pfam-A** E-value cut-off to **1.0**

This is the **sequence** that you submitted:

```
MKRVLKLLFMIVAFMTAVFAGSGQASADSTTGITQDIHIHISLGLNLPSGTDVTKLRAPTFEIYDISDQFNEA
DDPKEFTAKFPLGGQSYAKNFIEKHSLKPLSRQTGNKVNSSIDFIVPGCDAYLIVQTDENGVIENAGNNGTFT
LPFVFLMNDDFKIDDQGLMWFQIKGKTSLVQRSAYFFKYGKNAGGELPLSDAKFVLYRLDGSIKLYCTNNGGF
KASASPLSDDEIAKFTSNSAGLVMYDRESLDSGTYYFSEVQAPKGYRITDEARKIEVYIPKKLSDGVKVNGTA
LEELYDQKLSDGAVSAAKPRIYNYSIENPPSNSGKTNTPGKPNTPGKPNTSGKPTTPNSVRKKGLWGMLPQTG
EAKSIMALLGIGIICLVVLVSVGRRNYKEEH
```

You can bookmark this URL to retrieve your results later:

http://pfam.xfam.org/search/sequence/results/8A550A80-E998-11E4-ADCF-D176298E2F76

Return to the search form to look for Pfam domains on a new sequence.

Significant Pfam-A Matches

Show or hide all alignments.

Family	Description	Entry type	Clan	Envelope		Alignment		HMM		HMM length	Bit score	E-value	Predicted active sites	Show/hide alignment
				Start	End	Start	End	From	To					
Cna_B	Cna protein B-type domain	Family	CL0287	195	278	196	270	2	55	70	25.4	8.6e-06	n/a	Hide

```
#HMM       eGaefkLldeggkvvgke....................tlttdanGkytfk..nLppGnYtlkEvkaPaGYkltdd
#MATCH     ++a+f L+  +g+ +  +                    + t+++ G +  +   L++G+Y + Ev+aP+GY +td+
#PP        5666666666666655.56666666666788888888889999999876652268******************997
#SEQ       SDAKFVLYRLDGSIKL-YctnnggfkasasplsddeiaKFTSNSAGLVMYDreSLDSGTYYFSEVQAPKGYRITDE
```

C

Sequence search results

Show the detailed description of this results page.

We found **1** Pfam-A match to your search sequence (**all** significant). You did not choose to search for Pfam-B matches.

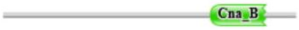

Hide the search options and sequence that you submitted.

You ran **one** job, a **Pfam-A** search with **ID** 5FE98356-E999-11E4-B984-E44495374F7B.

These are the options that you chose:

- You set the **Pfam-A** E-value cut-off to **1.0**

This is the **sequence** that you submitted:

```
MKNHKKLRNALATLLLALPLALQGAVGVKTAQAAETSTETATVTLHKYVFDKSLPSDKIDNSKSQDEINAWLT
KNNAEALDGVEFTAYDVTSEYADAYKTATGDKNESPADAAKTASAAVAKKADALQKTATVVGKQTTANGGLAS
FANLPLRDANGNYKAYLFAETDAPANITQKAEPFVLAMPIYGADGKTVQKSINIYPKNVKQSDKKTLNDNRSH
HDFTAGEKINYSIETVVPWNIANKKVYTITDNPSKGLIMDADTIQIEGLASNKYTVKKNADNGFTITIPAANL
AAFAGKTLKTTVKGHLSIEDLTLIDTGIPNKATAKVDNEAHHEVKSEEVFTGGKKFVKVDGSNQSKTLAGAQF
QLVIVKNGQVVKYAHGNEKDGYTFDTNNTNVATKTTGENGQFEFAGLKYSESLEAGESYAVKEVKAPTGYDLL
KDPVLFTVTKDSYKTVQAADGQKISNTKKGGFLPSTGGMGIVLFIAAGVVVMAGAAGTMIVRRNRRENI
```

You can bookmark this URL to retrieve your results later:

http://pfam.xfam.org/search/sequence/results/5FE98356-E999-11E4-B984-E44495374F7B

Return to the search form to look for Pfam domains on a new sequence.

Significant Pfam-A Matches

Show or hide all alignments.

Family	Description	Entry type	Clan	Envelope		Alignment		HMM		HMM length	Bit score	E-value	Predicted active sites	Show/hide alignment
				Start	End	Start	End	From	To					
Cna_B	Cna protein B-type domain	Family	CL0287	360	455	360	454	1	69	70	51.8	4.7e-14	n/a	Hide

```
#HMM       leGaefkLldeggkvvgke...................tlttdanGkytfknL......ppGn.YtlkEvkaPaGYkltddpteftitensdvkv
#MATCH     l+Ga+f+L+ + ++ v+k+                   t tt++nG+++f +L      + G+ Y +kEvkaP+GY l +dp+ ft t++s ++v
#PP        89**************99************************************************************************98776
#SEQ       LAGAQFQLVIVKNGQVVKYahgnekdgytfdtnntnvaTKTTGENGQFEFAGLkyseslEAGEsYAVKEVKAPTGYDLLKDPVLFTVTKDSYKTV
```
