# Supplementary material for: Human Gut-Commensalic Lactobacillus ruminis ATCC 25644 Displays Sortase-Assembled Surface Piliation: Phenotypic Characterization of Its Fimbrial Operon through In Silico Predictive Analysis and Recombinant Expression in Lactococcus lactis
Source: PLoS One. 2015 Dec 28;10(12):e0145718. doi: 10.1371/journal.pone.0145718 (PMC4692528; doi:10.1371/journal.pone.0145718)
Supplement: S3 Fig — Shown is a multiple sequence alignment of 91 nucleotides (nt) that lie directly upstream from the first five codons of the lrpC gene (the first gene in the L. ruminis fimbrial lrpCBA operon). DNA sequence (106 nt) covering this region was taken from the genomes of the L. ruminis ATCC 25644, SPM0211, ATCC 27782, DPC 6832, and GRL1172 strains and aligned using MultAlin [58] (http://multalin.toulouse.inra.fr/multalin/multalin.html). Those nucleotides that are common to all sequences in the alignment, and thus that make up the consensus sequence, are denoted in red. Other nucleotides that are not part of the consensus sequence are denoted in black. (PDF) [file pone.0145718.s003.pdf]

```
           1        10        20        30        40        50        60        70        80        90        100   106
           |--------+---------+---------+---------+---------+---------+---------+---------+---------+---------+-----|
ATCC25644  CGAAGAGAGGTTGTCTGATGACGGTATCTTTCAGACGTTATGTTTGGAGTATTTGTTGGTTAGTGATAGATAAGAATGGAGAGCAACTGATATGGAAAGAAATAAA
  SPM0211  CGAAGAGAGGTTGTCTGATGACGGTATCTTTCAGACGTTATGTTTGGAGTATTTGTTGGTTAGTGATAGATAAGAATGGAGAGCAACTGATATGGAAAGAAATAAA
ATCC27782  CGAAGAGAGGTTGTCTGATGACGGTATCTTTCAGACGTTATGTTTGAAGTATTTGTTGGTTAGTGATAGATAAGAATGGAGAGCAACTGATATGGAAAGAAATAAA
  GRL1172  CGGAGAGAGGTTGTCTGATGACGGTATCTTTCAGACGTTATGTTTGAAGTATTTGTTGGTTAGTGATAGATAAGAATGGAGAGCAACTGATATGGAAAGAAATAAA
  DPC6832  CGAAGAGAGGTTGTCTGATGACGGTATCTTTCAGGCGT-ATGCTTGGAGTATTTGTTGGTTAGTGATAGATAAGAATGGAGAGCAACTGATATGGAAAGAAATAAA
Consensus  CG.AGAGAGGTTGTCTGATGACGGTATCTTTCAG.CGT.ATG.TTG.AGTATTTGTTGGTTAGTGATAGATAAGAATGGAGAGCAACTGATATGGAAAGAAATAAA
```
