# Supplementary figures and images for: Human Gut-Commensalic Lactobacillus ruminis ATCC 25644 Displays Sortase-Assembled Surface Piliation: Phenotypic Characterization of Its Fimbrial Operon through In Silico Predictive Analysis and Recombinant Expression in Lactococcus lactis
Source: PLoS One. 2015 Dec 28;10(12):e0145718. doi: 10.1371/journal.pone.0145718 (PMC4692528; doi:10.1371/journal.pone.0145718)

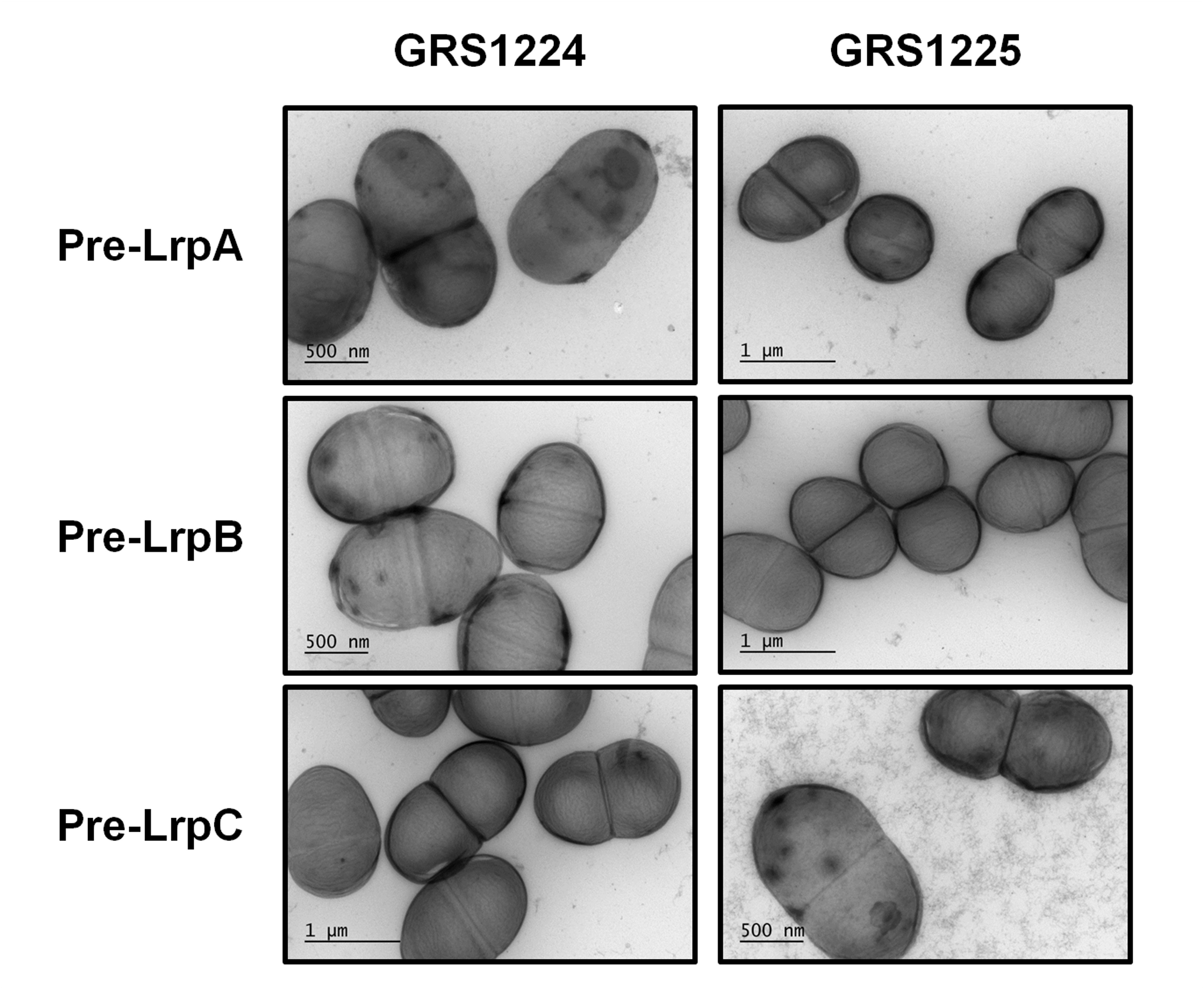

Supplement: S4 Fig — Immunogold labeling and electron microscopy of the WT GRS1224 (panels to the left) and LrpC pilin-deleted GRS1225 (panels to the right) lactococcal clones were carried out with preimmune serum as described in Materials and Methods. Preimmune serum of the same rabbit from which each pilin-specific antiserum had been raised was used for these experiments. Nisin-induced recombinant lactococci were single-labeled using the preimmune sera, Pre-LrpA (undiluted; top panels), Pre-LrpB (diluted 1:4; middle panels), and Pre-LrpC (diluted 1:8; bottom panels), along with protein A-10-nm gold particles. Representative EM images are shown. Scale bars are included in each panel. (TIF) [file pone.0145718.s004.tif]

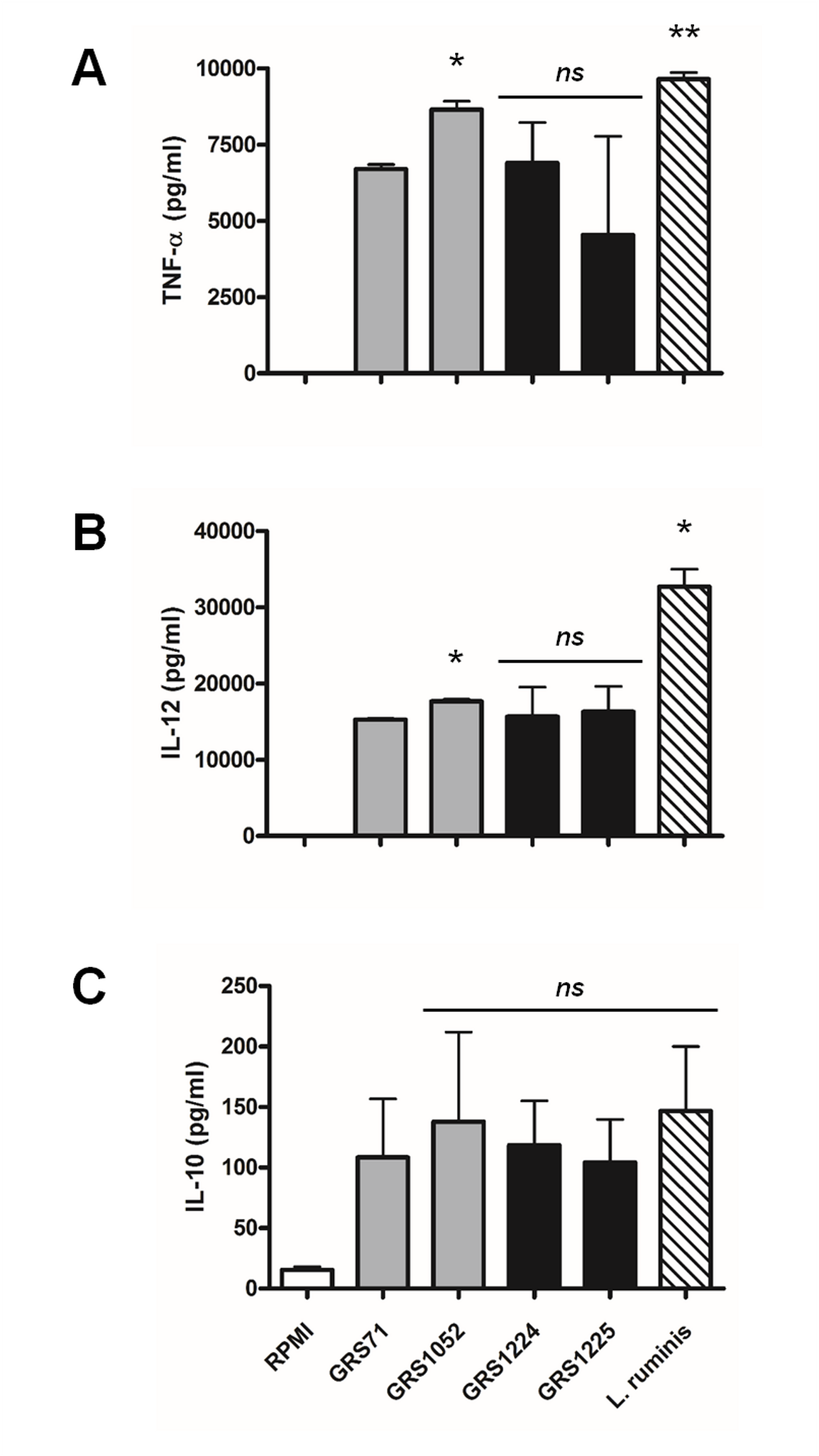

Supplement: S5 Fig — Human monocyte-derived dendritic cells (moDCs) were exposed to L. ruminis cells or the recombinant WT (GRS1224) and LrpC-deleted (GRS1225) lactococci and the subsequent stimulated production of the TNF-α (A), IL-12 (B), and IL-10 (C) cytokines was then measured using the same protocol from our previous published studies [11,14]. For this, overnight grown cultures were prepared (for details, see Materials and Methods) and then normalized according to OD600 in RPMI 1640 medium that contained 10% FCS, antibiotics, L-glutamine, and HEPES, but had no interleukin (IL)-4 and granulocyte-macrophage colony-stimulating factor (GM-CSF). Bacteria and moDCs were combined with a MOI of 50 and then incubated for about 24 hours at 37°C with 5% CO2 content. Cell culture supernatants were recovered and an estimation of the cytokine levels made by ELISA measurements using the BD OptEIA™ ELISA kit (BD Biosciences). The moDCs used in the experiments were generated from the blood of two different donors and then readied using essentially the same method as before [11,14]. GRS71 and GRS1052 cells, as well as RPMI cell-culture medium, were used as controls and treated as mentioned above. Triplicate measurements were made for each experiment, with limit bars showing the SEM. Statistical differences for individual pairwise comparisons against the GRS71 control are specified as ** = P ≤ 0.01 (very significant), * = P ≤ 0.05 (significant), or ns = P > 0.05 (not significant). (TIF) [file pone.0145718.s005.tif]
